# MicrobesFlux: a web platform for drafting metabolic models from the KEGG database

**DOI:** 10.1186/1752-0509-6-94

**Published:** 2012-08-02

**Authors:** Xueyang Feng, You Xu, Yixin Chen, Yinjie J Tang

**Affiliations:** 1 Department of Energy, Environmental and Chemical Engineering, Washington University in St. Louis, Saint Louis, MO 63130, USA; 2 Department of Computer Science and Engineering, Washington University in St. Louis, St. Louis, MO, 63130, USA; 3 Energy Biosciences Institute, University of Illinois, Urbana, IL 61801, USA

## Abstract

**Background:**

Concurrent with the efforts currently underway in mapping microbial genomes using high-throughput sequencing methods, systems biologists are building metabolic models to characterize and predict cell metabolisms. One of the key steps in building a metabolic model is using multiple databases to collect and assemble essential information about genome-annotations and the architecture of the metabolic network for a specific organism. To speed up metabolic model development for a large number of microorganisms, we need a user-friendly platform to construct metabolic networks and to perform constraint-based flux balance analysis based on genome databases and experimental results.

**Results:**

We have developed a semi-automatic, web-based platform (MicrobesFlux) for generating and reconstructing metabolic models for annotated microorganisms. MicrobesFlux is able to automatically download the metabolic network (including enzymatic reactions and metabolites) of ~1,200 species from the KEGG database (Kyoto Encyclopedia of Genes and Genomes) and then convert it to a metabolic model draft. The platform also provides diverse customized tools, such as gene knockouts and the introduction of heterologous pathways, for users to reconstruct the model network. The reconstructed metabolic network can be formulated to a constraint-based flux model to predict and analyze the carbon fluxes in microbial metabolisms. The simulation results can be exported in the SBML format (The Systems Biology Markup Language). Furthermore, we also demonstrated the platform functionalities by developing an FBA model (including 229 reactions) for a recent annotated bioethanol producer, *Thermoanaerobacter sp*. strain X514, to predict its biomass growth and ethanol production.

**Conclusion:**

MicrobesFlux is an installation-free and open-source platform that enables biologists without prior programming knowledge to develop metabolic models for annotated microorganisms in the KEGG database. Our system facilitates users to reconstruct metabolic networks of organisms based on experimental information. Through human-computer interaction, MicrobesFlux provides users with reasonable predictions of microbial metabolism via flux balance analysis. This prototype platform can be a springboard for advanced and broad-scope modeling of complex biological systems by integrating other “omics” data or ^13^ C- metabolic flux analysis results. MicrobesFlux is available at http://tanglab.engineering.wustl.edu/static/MicrobesFlux.html and will be continuously improved based on feedback from users.

## Background

Arising interests in metabolic engineering have focused on systems analyses of cell metabolisms [1-6]. Metabolic flux analysis is a key approach in systems biology that determines the *in vivo* enzyme activities in a metabolic network and links genotype to phenotypes. In the past decade, over 100 genome-scale metabolic models have been constructed for *E.coli*[7-9], *Bacillus subtilis*[10,11], and *Saccharomyces cerevisiae*[12-14] to expand our understanding of their physiologies. Although metabolic model reconstructions are important, their development is still much slower than that of high throughput genome sequencing of diverse microorganisms [15] due to three reasons. First, constructing metabolic models is normally a slow, tedious and labor-intensive process, including over 90 steps from assembling genome annotations of target organisms to validating the metabolic model by various “omics” studies [16]. Second, a systemic reconstruction of metabolic models often relies on commercial software (e.g. MATLAB) and requires proficient programming skills. Though the majority of microbiologists know the physiology of environmental microorganisms well, they may not have practical experience with computer modeling. Third, for studying numerous poorly understood environmental organisms, it is necessary to build a programming-free and user-friendly computer platform that facilitates researchers to convert a vast amount of experimental data into model constraints to reduce solution space and to improve the model predictability.

Currently, a few software tools are available to assist biologists in metabolic modeling. SimPheny (http://www.genomatica.com) is a commercial software tool for genome-scale Flux Balance Analysis (FBA). Webcoli supplies diverse approaches for users to reconstruct a genome-scale *E.coli* metabolic model [17]. OpenFLUX is a computationally efficient software tool for ^13^ C-assisted metabolic flux analysis [18]. OptFlux is an open-source, modular software package for FBA and microbial strain design using an evolutionary optimization algorithm [19]. BioMet Toolbox provides web-based resources for FBA and transcriptome analysis [20]. Model SEED [21] can automatically generate genome-scale metabolic models for different microbes based on the RAST (Rapid Annotation using Subsystem Technology) annotations. FAME [22] is a web-based modeling tool for creating, editing and analyzing metabolic models for microorganisms from the KEGG database. To augment these tools, we are developing MicrobesFlux, a web platform to draft and reconstruct metabolic models (Table1). This system has several distinguishing features: 1) it can automatically generate metabolic models of ~1,200 microbes sequenced in the KEGG database (http://www.genome.jp/kegg/), 2) it allows users to fine tune the metabolic models according to user-defined requests, and 3) it can help researchers perform both flux balance analysis (FBA) with user-defined objective functions and dynamic flux balance analysis (dFBA). The marriage of flux model generation and customized model reconstruction is of great benefit to biologists since they can easily validate or refute hypotheses in microbial metabolism by drafting and comparing numerous metabolic models. In the future, this prototype platform will potentially be able to interact with other software packages (e.g. OptFlux [19], COBRA [23]) to perform broad-scope metabolic modeling of complex biological systems. 

**Table 1:** Comparison of MicrobesFlux and other web-based fluxomics software

		**MicrobesFlux**	**FAME**	**Model SEED**	**Webcoli**	**BioMet Toolbox**
**Model generation**	Database	KEGG	KEGG	RAST	iJR904	None
	Number of organisms	1,194	~780	~5,000	1	NA
**Embedded functions for model reconstruction**^**1**^	Inflow/outflow introduction	●	●		●	
	Biomass production implementation	●	●	●	●	
	Automated generation of biomass composition^2^			○		
	Heterologous pathways	●	●		●	
	Knock out pathways	●	●		●	
	Reactant/product Modification	●	●			
	Automated mass balance	●	●	●	●	●
	Automated compound charging and charge balance			●		
	Transport reactions with coupling to ATP and proton translocation^3^	○	○		○	
	Prediction of reaction directionality/reversibility based on thermodynamics^4^					
	Gap-fill^5^	○	○	○		
	Gene-Protein-Reaction associations			●	●	
	Reaction compartments		●	●		
**Flux analysis**	FBA	●	●	●	●	●
	FBA with customized objective function	●				●
	Dynamic FBA	●				
	Flux Variability Analysis		●			
**Output**	Pathway visualization	●	●	●	●	●
	SBML output	●	●	●	●	●

1: The embedded functions, as developed in MicrobesFlux, FAME and Webcoli, are the functional modules that are directly incorporated into the web-based software to provide human-computer interaction and to minimize users’ programming work. Model SEED can achieve the model reconstruction via manual programming on a SBML-formatted metabolic model, instead of using the embedded functions [22].

2: The biomass composition needs to be manually inputted in MicrobesFlux and FAME. Model SEED can automatically generate one template biomass composition for different organisms. Webcoli has fixed the biomass composition of *E.coli* in the system.

3: The transport reactions (i.e., inflow/outflow) can be coupled to ATP and proton translocation manually in MicrobesFlux, FAME, and Webcoli.

4: None of the software included in Table1 predicts the reaction directionality/reversibility based on thermodynamics.

5: Gap-fill is achieved manually in MicrobesFlux and FAME. Model SEED uses a computational algorithm to achieve semi-automatic gap-fill.

## Implementation

**Figure 1:**
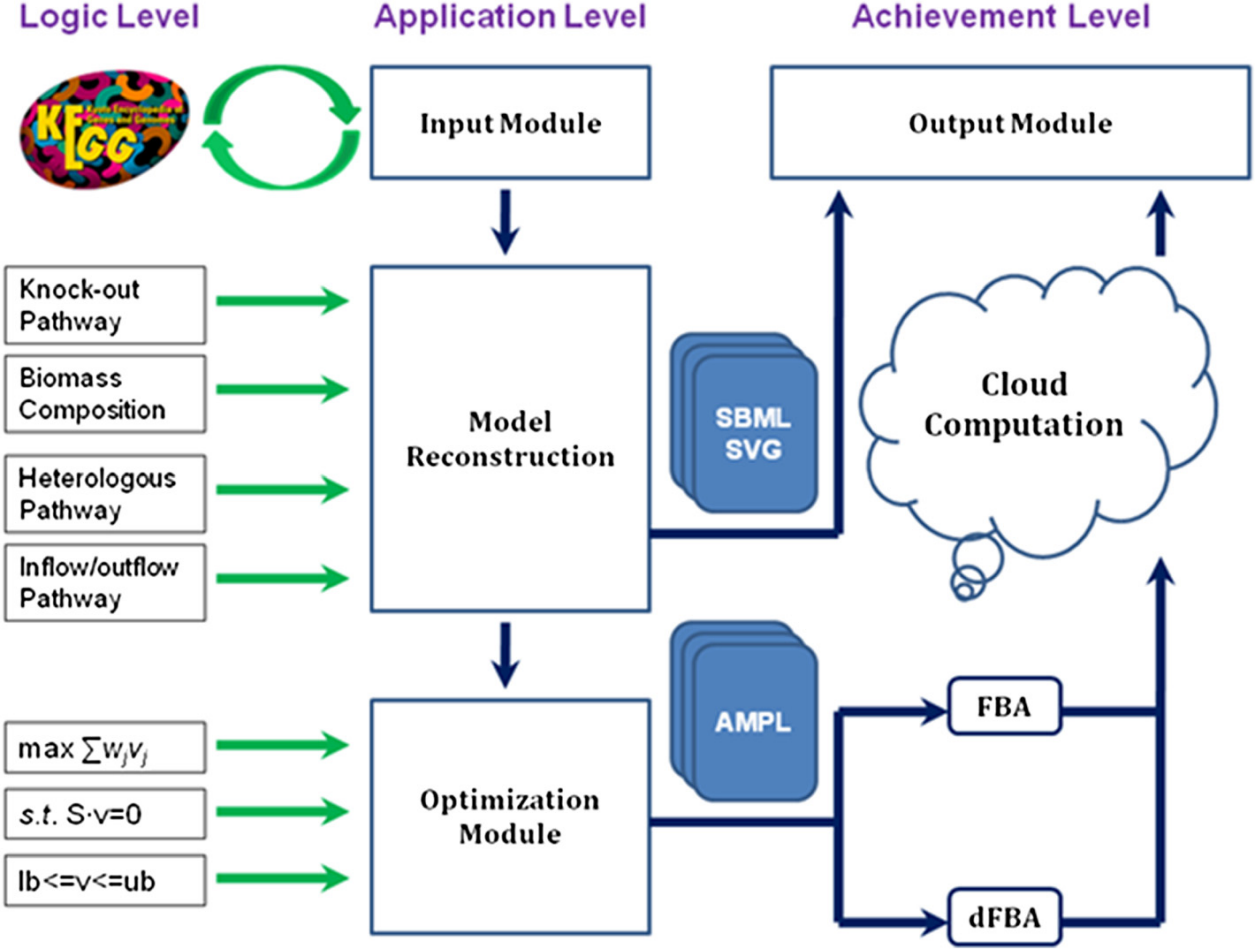
Architecture of MicrobesFlux.

MicrobesFlux is an open-source platform that is free to academic users with mandatory registration. It has three high-level components: the *logic level*, the *application level*, and the *achievement level*. The *logic level* includes KGML and KEGG LIGAND, two fundamental databases used in MicrobesFlux. KGML is for organism-specific metabolic networks and KEGG LIGAND is for general enzymatic reactions and metabolites. The basic principles for metabolic model reconstruction and constraint-based flux analysis are summarized in the logic level (Figure1). In the *application level*, organism-specific metabolic networks are downloaded from the KEGG database to generate metabolic models, which are then used for customized reconstruction. The reconstructed metabolic network can be formulated as either an FBA or a dynamic FBA model to determine the flux distributions under metabolic steady or dynamic states, respectively. The constraint-based flux analysis will be accomplished in the *achievement level*, by using state-of-the-art optimization solvers, such as IPOPT (Interior Point OPTimizer). The calculated flux distributions and the reconstructed metabolic network are recorded in the SBML format (The Systems Biology Markup Language) and the metabolic networks can be viewed in a web browser using Scalable Vector Graphics (SVG) format. Both results are sent to users via email in the output module. The front end of MicrobesFlux is written with Google Web Toolkit technology and the backend is written in Python with the Django web framework. In summary, three key features to facilitate flux model development are embedded in MicrobesFlux: 1) high-throughput metabolic models generation; 2) customized metabolic models drafting; and 3) constraint-based flux analysis in steady and dynamic metabolic states.

### High-throughput generation of metabolic model

MicrobesFlux can serve as a platform to build metabolic models for 1,194 organisms sequenced in the KEGG database. To generate a metabolic model for a specific microorganism, the gene annotation information is downloaded from a set of corresponding KGML files, and cross-referenced with the KEGG LIGAND database. The generated pathway network serves as a seed model subject to further customization by the users.

### Customized drafting of metabolic models

The model draft of the designated microorganisms, automatically generated from the KEGG database, should be reconstructed to fill in the gaps between the genome annotation and the functional metabolism. MicrobesFlux can provide a list of tools to help users build customized metabolic models. They include: 1) the introduction of inflow/outflow fluxes to describe the nutrient transport between the cytosol and the extracellular medium; 2) the implementation of biomass production reactions with the manual identification of the organism-specific biomass composition; 3) the inclusion of heterologous pathways or gap-fill pathways that do not exist in the seed model; and 4) the modification of metabolic pathways by tuning reaction directions and reactants/products species in the reactions. The functions in MicrobesFlux can help users build a drafted metabolic model for further reconstruction and flux analysis. The metabolic pathway network can be exported as SBML and viewed with SVG.

It is necessary to point out that the complete genome-scale metabolic model reconstructions cannot be automatically achieved in MicrobesFlux (Table1) yet. According to the protocol made by Thiele and Palsson [16], a high-quality genome-scale metabolic reconstruction requires 4 stages and 94 steps. By using MicrobesFlux, users can finish 100% of stage 1 (i.e., draft recognition, 5 out of 5 steps), 69% of stage 2 (i.e., refinement of reconstruction, 22 out of 32 steps), 100% of stage 3 (i.e., conversion of reconstruction into a computable format, 5 out of 5 steps) and 33% of stage 4 (i.e., network evaluations, 17 out of 52 steps). More specifically, several iterative model curation steps in stage 2 and 4 cannot be achieved in the current version of MicrobesFlux yet. For example, the compound charging (step 8 in stage 2) and charge balance in metabolic reactions such as electron transport chains (step 20 in stage 2) cannot be automatically curated due to the lack of information in the KEGG LIGAND database and KGML files used for building the metabolic models in MicrobesFlux. In addition, the automated computational gap-analyses (step 46–48 in stage 4) and gap-fill algorithms (step 51–58 in stage 4) are not incorporated in MicrobesFlux, although, for a medium-scale metabolic model, users can manually identify gaps (such as dead-end metabolites) and adjust the metabolic reactions to fill these gaps. Other features that are essential for completing a high-quality genome-scale metabolic model but are not covered by MicrobesFlux include predicting reaction directions based on thermodynamics (step 10), assigning the location of genes and metabolic reactions (step 11), and correlating genes, proteins and metabolic reactions (step 13). In order to construct an accurate genome-scale metabolic model, these steps need to be done by users off-line to expand the MicrobesFlux-drafted model. Given this, the MicrobesFlux software should be used as a toolkit for “drafting” instead of “finalizing” metabolic models.

### Constraint-based flux analysis in steady and dynamic metabolic states

For each reconstructed metabolic network, users can choose to perform either FBA or dFBA tasks to determine flux distributions under metabolic steady or dynamic states. The objective functions used in constraint-based flux analysis can either be the commonly used “maximizing biomass”, or a user-defined objective function. The users can also define the upper and lower bounds for each flux in FBA and dFBA. MicrobesFlux converts the constraint-based flux analysis model on the reconstructed network to a large-scale optimization problem with constraints, and employs IPOPT (an optimization solver based on an interior-point algorithm) to find an optimal solution [24]. The solutions acquired by IPOPT are then transformed into the metabolic fluxes as requested by users. MicrobesFlux automatically distributes these large-scale optimization tasks to a cluster of machines so that multiple users can conduct flux analyses on our system simultaneously. Our system also supports the dynamic simulation of metabolic fluxes by using the static optimization approach (SOA) [25]. In this approach, the users can assume that the entire dynamic microbial metabolism is composed of numerous pseudo-steady states. For each pseudo-steady state, a conventional FBA problem is formulated with the user-defined inflow and outflow fluxes. In other words, a dFBA problem will be converted to multiple mini-FBAs that are subject to constraints from the measurement of time-dependent inflow (substrate uptake) and outflow fluxes (metabolite production). To avoid extensive analytical efforts to measure inflow and outflow fluxes at each time interval, the users can use an empirical or a kinetic model to estimate the time-dependent inflow/outflow fluxes for the mini-FBAs through an entire growth period based on limited measurement data. The dynamic flux simulation is of particular industrial interest [26], since many biological systems cannot maintain a meaningful metabolic steady state during the fermentation process.

## Results and discussion

**Figure 2:**
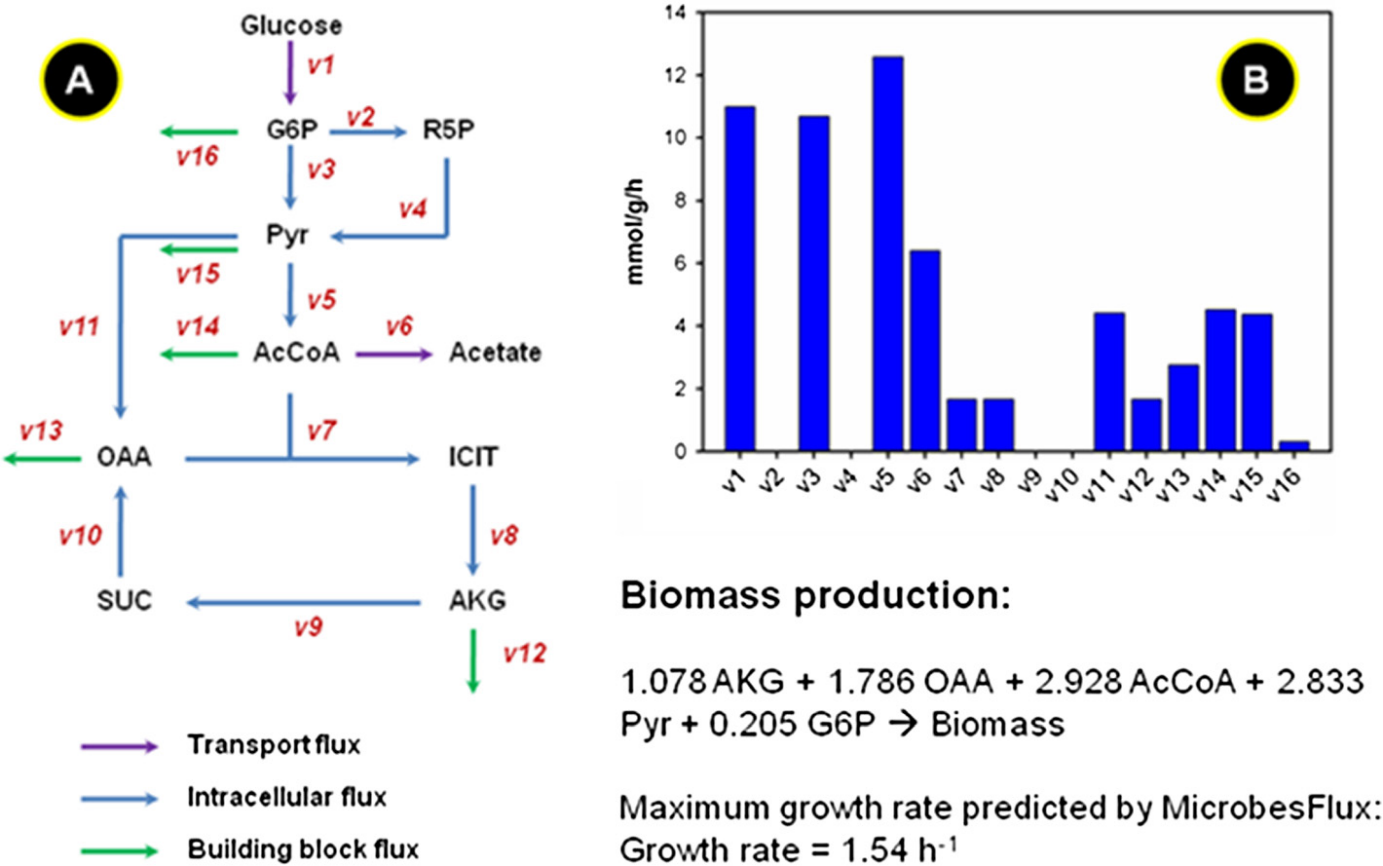
(**A**) **Pathway network of the TOY model used in MicrobesFlux, and (B) the simulated flux distribution of the TOY model used in MicrobesFlux.** The same results were obtained by using “linprog” in MATLAB.

We have applied MicrobesFlux to a few case studies in drafting metabolic models. We first drafted a TOY model (Figure2), which has 10 metabolites and 16 fluxes, as a demonstration of the MicrobesFlux workflow. We then constructed a medium-scale stoichiometric model with 196 metabolites and 229 reactions for *Thermoanaerobacter* sp. strain X514, a thermophilic bacterium that is of great interest in cellulosic ethanol production [27]. The functionality and applicability of MicrobesFlux have been proved in both case studies.

### Case study 1: A toy model

**Figure 3:**
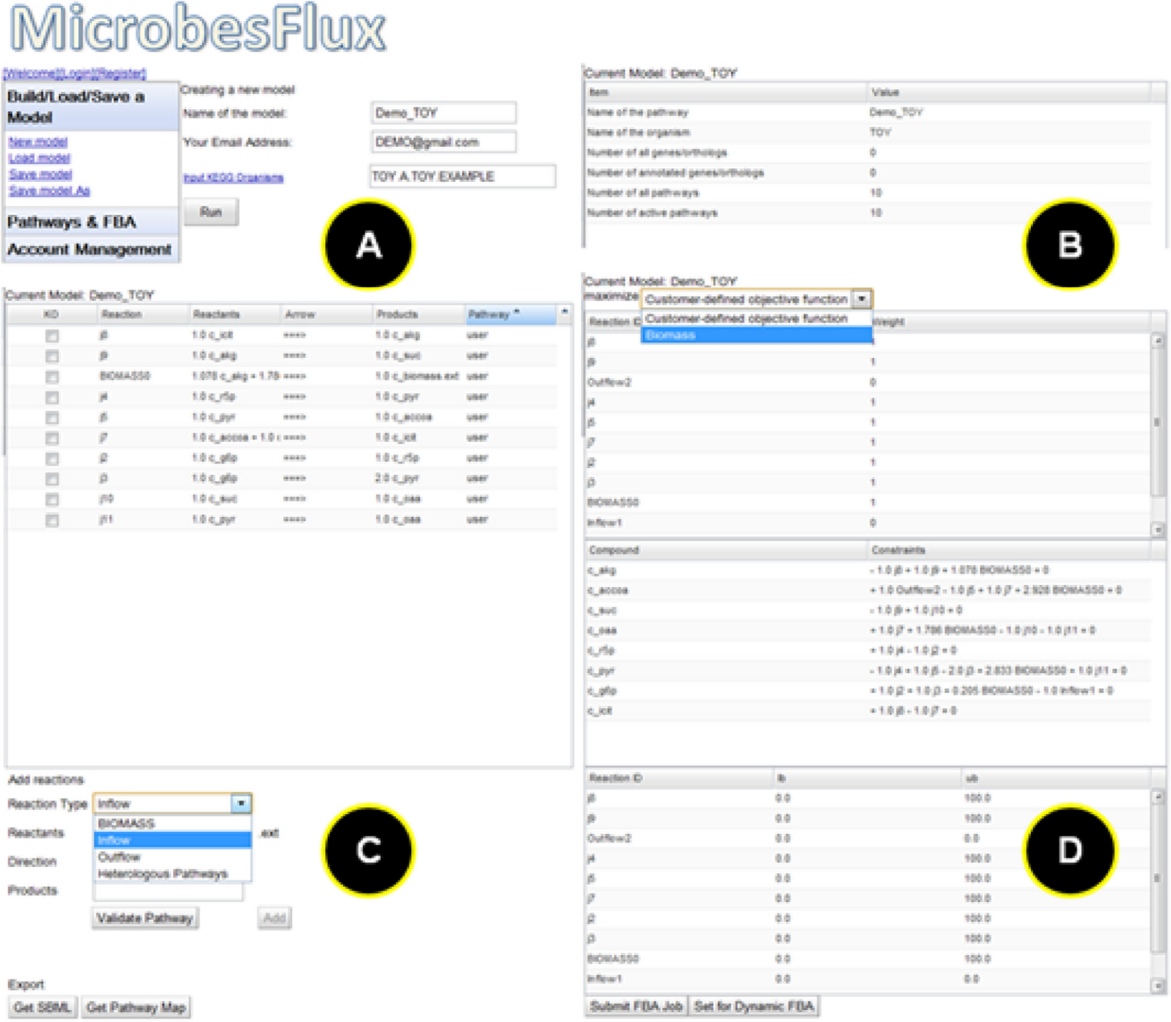
**Screenshot of the reconstruction of the TOY model by using MicrobesFlux.** (**A**) the TOY model loaded from MicrobesFlux; (**B**) the pathway information of the TOY model; (**C**) the customized reconstruction of the TOY model; and (**D**) the constraint-based flux balance analysis of the TOY model.

To demonstrate the use of the MicrobesFlux platform, a simple toy model was constructed, which only included the central metabolic pathways: the glycolysis pathway, the pentose phosphate pathway, the TCA cycle, and the anaplerotic pathway. Glucose represented the carbon substrate and acetate represented the extracellular metabolite product. The TOY model was loaded from MicrobesFlux (Figure3), which included 10 reactions that described the intracellular fluxes and lumped biomass production. Subsequently, the toy model was reconstructed by introducing the inflow flux: “Glucose → G6P” and the outflow flux: “AcCoA → Acetate”. The drafted TOY model was then used for constraint-based flux analysis by setting the objective function as “maximizing biomass” and fixing the inflow and outflow fluxes as 11.0 and 6.4 mmol/g DCW/h respectively. The reconstructed pathways and the simulated flux distributions from the TOY model can be found in Additional file 1 and Additional file 2. The simulated results from MicrobesFlux were confirmed by an independent linear optimization of the same TOY model via “linprog” in MATLAB (Additional file 3).

### Case study 2: A medium-scale metabolic model for *Thermoanaerobacter* sp. strain X514

Based on a similar flowchart to that of Case Study 1, we drafted a medium-scale metabolic model of *Thermoanaerobacter* sp. strain X514. *Thermoanaerobacter* species are thermophilic bacteria that can simultaneously convert both pentose and hexose to ethanol with high yield [28,29]. It can also be co-cultured with cellulosic *Clostridium thermocellum* species to produce ethanol from cellulose. *Thermoanaerobacter* sp. strain X514 was not sequenced until recently. The metabolic pathways of the central metabolism have been studied via a ^13^ C-based metabolic pathway analysis [28]. However, there is limited experimental data on the secondary metabolism, including the peripheral pathways of *Thermoanaerobacter* sp. strain X514. Therefore, this study chose to reconstruct a medium-scale model that included only the central metabolic pathways to demonstrate the applicability of MicrobesFlux-based FBA for understanding strain X514’s ethanol metabolism.

Using the experimental data of strain X514 reported in [28] and [29], we applied MicrobesFlux and reconstructed a metabolic model that described the carbohydrate metabolism and amino acids biosynthesis in strain X514. The drafted model consisted of 196 metabolites and 229 reactions, of which 162 reactions were intracellular reactions, 19 reactions were inflow/outflow reactions, 39 reactions were gap-filling reactions, and 9 reactions were used for biomass building block syntheses and biomass production. The intracellular reactions in the drafted model were derived from the genome-annotation of *Thermoanaerobacter* sp. strain X514 from the KEGG database, considering only the carbon and cofactor balance. The inflow/outflow reactions were introduced into the metabolic model only if the transporters of the specific substrates or extracellular products had been reported by published research. Moreover, two algorithms were employed to fill the gaps in the metabolic pathways. First, we implemented un-annotated pathways identified from the ^13^ C-assisted pathway analysis (e.g. the Re-type citrate synthase in TCA cycle) into the metabolic model of strain X514 [28]. Since the draft model was a simplified model focusing on carbohydrate metabolism and amino acids biosynthesis, the gaps might have been generated due to the lack of consideration of the metabolites exchange between the pathways that were included in the model (e.g. carbohydrate metabolism) and other pathways that were not included in the model (e.g. purine metabolism). Accordingly, we employed the principle of introducing the metabolite-exchange-reactions to fill the gaps. For example, UTP is involved in both carbohydrate metabolism and RNA synthesis. Since we did not include the RNA synthesis pathway in the MicrobesFlux model, we instead introduced a UTP exchange pathway to fill the gap. The gap-filling reactions were evaluated carefully to make sure that each one of them was necessary for feasible predictions of biomass production.

**Figure 4:**
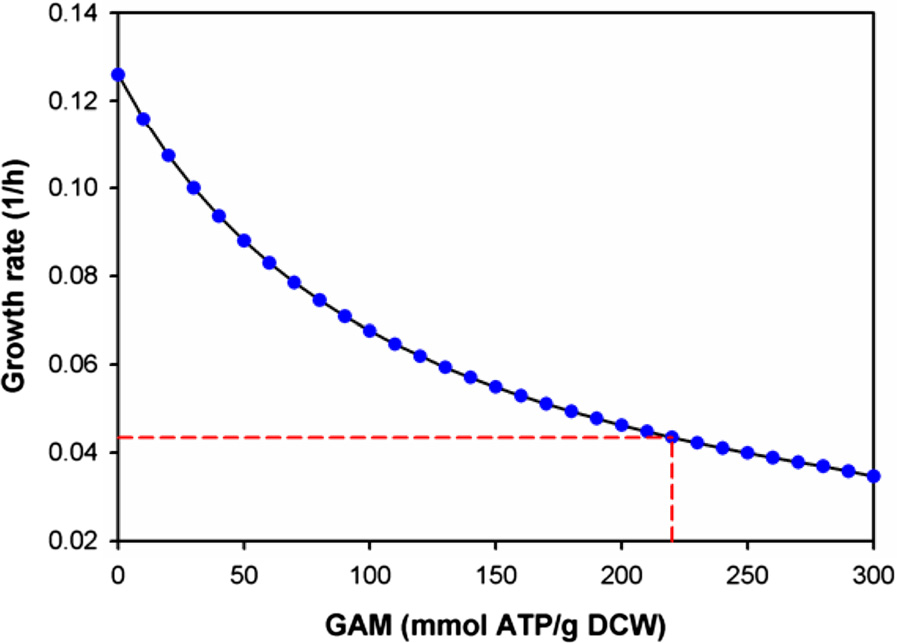
**Estimation of the growth-associated maintenance (GAM) in *Thermoanaerobacter* sp. strain X514.** From this comparison, the GAM value (red dotted line) that was consistent with the experimental data (i.e. growth rate was 0.042 h^-1^[29]) could be estimated and was indicated on the figure as a 220.0 mmol ATP/g DCW.

**Figure 5:**
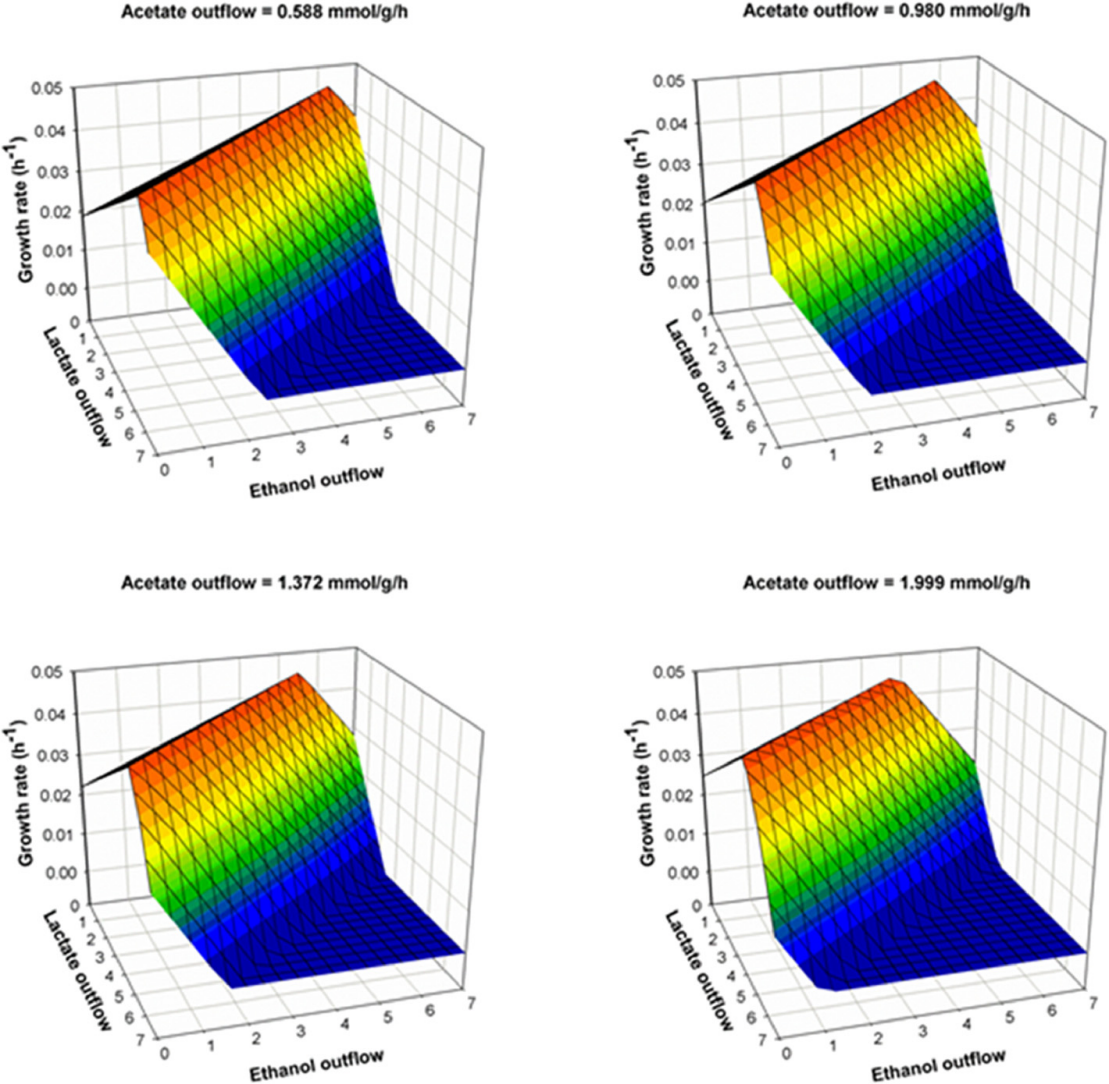
**Predictions of the relationship between growth rate and outflow fluxes [Unit: mmol/g DCW/h] in *Thermoanaerobacter* sp. strain X514.** The glucose inflow flux was fixed as 3.92 mmol/g DCW/h.

The biomass composition of *Thermoanaerobacter* sp. strain X514 is not yet available. In the drafted model, we used the reported biomass composition of a related species, *Clostridium acetobutylicum*[30]. Besides the biomass composition, the growth-associated maintenance (GAM) and non-growth associated maintenance (NGAM) energies were found to play an important role in simulating the growth rate of organisms [30-32]. In our model simulation, the NGAM was chosen as 7.6 mmol ATP/g DCW/h, as reported previously [32]. To identify the GAM, we plotted the relationship between growth rate and GAM (Figure4), and found the value of GAM that could match the experimental measurement (i.e. growth rate of strain X514 as 0.042 h^-1^ reported in [29]) to be 220.0 mmol ATP/g DCW. This fitted value is higher than the previously reported GAM value (150.0 mmol ATP/g DCW) in a thermopilic cellulose degrader (*Clostridium thermocellum*) [33]. Such a high value of GAM in strain X514 could be associated with the much higher ethanol productivity in X514 than *C. thermocellum* during cell growth [34]. This result is consistent with the positive correlation of ethanol yield and maintenance-energy in other microbial species [34]. The strain 514 model and the simulated flux distributions can be found in Additional file 4 and Additional file 5. Based on this model, we also predicted the correlations between growth rate, ethanol production, and waste product outflow (Figure5). The prediction shows a trade-off relationship between ethanol production and growth rate. The ethanol production can be increased by 25% (i.e. increased from 6.3 mmol/g DCW/h to 7.8 mmol/g DCW/h) while halving the growth rate (i.e. decreased from 0.045 h^-1^ to 0.027 h^-1^). By inhibiting the acetate production from 2.0 mmol/g DCW/h to 0.6 mmol/g DCW/h, the ethanol production under the optimal growth conditions could be improved by 33%. Therefore, we have shown the platform is able to make reasonable predictions for the biomass growth in response to metabolites synthesis.

## Conclusion

MicrobesFlux is designed for semi-automatic and high-throughput drafting of metabolic models for environmental microorganisms based on genome annotations in KEGG. Unlike the model organisms that have been systemically studied via different “omics” approaches, the environmental organisms have more complex metabolic features and fewer measurements from laboratories. Therefore, MicrobesFlux is a platform to construct models for predicting cell metabolisms. This main goal of this software is to offer an interface to make flux modeling as simple and efficient as possible for users. On the other hand, the validation of a genome-scale metabolic model can be a challenge due to the requirement of manually tuned pathways and due to a lack of experimental data to confirm functional pathways. For example, *Thermoanaerobacter* sp. strain X514 (used in the MicrobesFlux case study) is capable of growing under high temperatures and converting sugars to ethanol, which can be simulated by a medium-scale metabolic model. Such a convenient FBA model has decent predictive power for *in silico* studies of a non-model environmental microorganism for bioethanol production at different growth rates (Figure5). However, many other metabolic features (e.g., secondary metabolisms) in the strain X514 are not fully understood yet. It requires extensive experimental studies, including transcriptomics and proteomics analyses, for a precise reconstruction of the genome-scale model. Although the X514 model in this study only focused on the central carbon metabolic pathways (<300 reactions), it serves as a good demonstration for users to learn how to use the MicrobesFlux platform to fill annotation gaps and reconstruct metabolic models for real applications.

In summary, we have developed MicrobesFlux to draft the metabolic models of environmental organisms from the KEGG database and to serve as a high-throughput tool for systems biology. The user manual is also provided along with this software (Additional file 6). MicrobesFlux relieves microbiologists from having to write computer programs for metabolic modeling. It covers a large number of sequenced organisms in the KEGG database and provides multiple approaches to assist systems biologists in model generation and reconstruction. Both FBA and dFBA (via SOA approach) can be done in MicrobesFlux to simulate the metabolic behaviors of organisms in metabolic steady state and dynamic state. The drafted model can also be used by other software packages for genome-scale model reconstruction and *in silico* predictions. In the future, we will implement broad fluxomic approaches (e.g. ^13^ C-metabolic flux analysis) in MicrobesFlux to improve the accuracy and predictive power of the drafted metabolic models.

## Availability and requirements

· **Project name:** MicrobesFlux

· **Project homepage:**http://tanglab.engineering.wustl.edu/static/MicrobesFlux.html

· **Operating systems:** Platform independent

· **Programming language:** Java and Python

· **License:** MicrobesFlux is freely available for noncommercial purposes.

· **Any restrictions to use by non-academics:** none

## Misc

Xueyang Feng and You Xu are the authors contributed equally to this work.

## Competing interests

The authors declare that they have no competing interests.

## Authors’ contributions

XF and YJT initiated this study. YX, XF and YC developed the MicrobesFlux software. XF and YX performed the flux analysis. XF and YJT wrote the manuscript. All authors revised and approved the final manuscript.

## Supplementary Material

Additional file 1 SBML of TOY model used in MicrobesFlux. Click here for file

Additional file 2 Simulated results of TOY model used in MicrobesFlux. Click here for file

Additional file 3 MATLAB Code for FBA of TOY model. Click here for file

Additional file 4 **SBML of drafted model of *Thermoanaerobacter* sp. strain X514.** Click here for file

Additional file 5 **Simulated results of drafted model of *Thermoanaerobacter* sp. strain X514.** Click here for file

Additional file 6 User manual of MicrobesFlux. Click here for file
