# Supplementary material for: MicrobesFlux: a web platform for drafting metabolic models from the KEGG database
Source: BMC Syst Biol. 2012 Aug 2;6:94. doi: 10.1186/1752-0509-6-94 (PMC3447728; doi:10.1186/1752-0509-6-94)
Supplement: Additional file 6 — User manual of MicrobesFlux. [file 1752-0509-6-94-S6.pdf]

MicrobesFlux: A Web Platform for Drafting Metabolic Models from the KEGG Database

Xueyang Feng and You Xu

May 20, 2012

1 Introduction

MicrobesFlux is a web-based platform for building, modifying, and analyzing metabolic models of multiple organisms. MicrobesFlux uses LIGAND database and KGML files from Kyoto Encyclopedia of Genes and Genomes (KEGG). MicrobesFlux is designed by Dr. Xueyang Feng and You Xu at Washington University in St. Louis.

MicrobesFlux uses the Google Web Toolkit for Web interface and uses the Django web framework for server logic and database management. Metabolic models are solved in MicrobesFlux by IPOPT (Interior Point OPTimizer), a nonlinear optimization solver.

1.1 Availability, licenses and contacts

MicrobesFlux is freely accessed via `http://tanglab.engineering.wustl.edu/static/MicrobesFlux.html`.
It supports modern Web browsers such as Google Chrome, Mozilla Firefox, or Safari. You are welcome to contact any of us for questions and comments:

Xueyang Feng, Ph.D.
Dept. of Energy, Environmental & Chemical Engineering,
Washington University in St. Louis
`fengx@seas.wustl.edu`
Tel: 314-348-6578

You Xu
Dept. of Computer Science & Engineering,
Washington University in St. Louis
`youxu@wustl.edu`
Tel: 314-935-9187

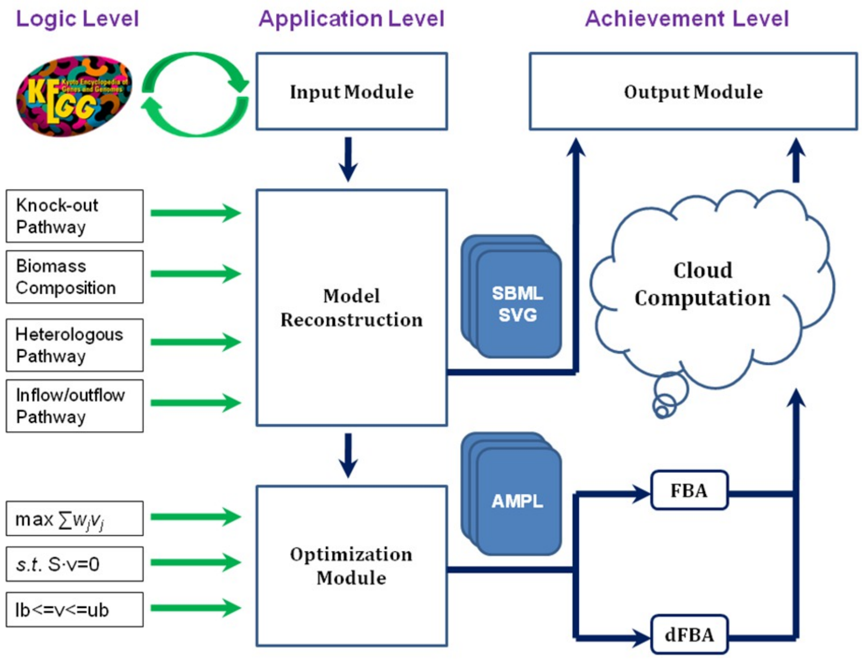

Figure 1: Architecture of MicrobesFlux

Yixin Chen, Ph.D.
Associate Professor, Dept. of Computer Science & Engineering,
Washington University in St. Louis
`chen@cse.wustl.edu`
Tel: 314-935-7528

Yinjie Tang, Ph.D.
Assistant Professor, Dept. of Energy, Environmental & Chemical Engineering,
Washington University in St. Louis
`yinjie.tang@seas.wustl.edu`
Tel: 314-935-3441

MicrobesFlux is also an open-source project. The source code of MicrobesFlux, in its entirety, is hosted on Google code. Users can report issues or request features using Google Code's issue tracking system: `http://code.google.com/p/kegg-dfba`.

1.2 Systems architecture

MicrobesFlux is designed with three high-level components: the logic level, the application level, and the achievement level (Figure 1).

In the logic level, the basic principles for metabolic model reconstruction and constraint- based flux analysis are summarized. In additional, the KEGG LIGAND database and KGML files are used as fundamental database in MicrobesFlux.

In the application level, organism-specific metabolic networks are loaded by reading KGML files of the designated organism. Metabolic reactions in the metabolic networks are cross-referred to KEGG LIGAND database. A seed metabolic model is generated and pursued for customized reconstruction and constraint-based flux analysis.

In the achievement level, a reconstructed metabolic model is formulated into an AMPL model files as either a problem, by designating the objective function and the boundary conditions of intracellular fluxes. We can conduct both flux balance analysis (FBA) or a dynamic FBA (dFBA) from the same metabolic model."

The FBA or dFBA problem is solved by IPOPT, a nonlinear optimization solver running on a cluster of computers. The achievement level can provide the reconstructed metabolic networks recorded in the SBML format (The Systems Biology Markup Language), and visualize the metabolic networks using the Scalable Vector Graphics (SVG) format. The calculated flux distributions are sent to users through email.

1.3 Caveats

When using MicrobesFlux to generate and reconstruct a metabolic model, please be aware that the reactions extracted from KEGG LIGAND database and KGML files are not always balanced. For example, the protons (H+, KEGG ID: C00080) sometimes will be absent in certain reactions. If the reactions imbalance is detrimental to the metabolic model, users do can **manually** curate the corresponding reactions in MicrobesFlux. However, automatic curation of un-balanced reactions in the KEGG database is beyond the current scope of MicrobesFlux. We plan to address it in the future versions.

Since the locations of metabolic reactions are not specified in KEGG LIGAND database or KGML files, the information about metabolic compartment (e.g., mitochondrial) cannot be assigned in the generated metabolic models.

2 Basic functions of MicrobesFlux

2.1 Welcome page

The landing page of MicrobesFlux (Figure 2) has five tabs that provide users the basic information of the functionality, architecture, and frequently asked questions about MicrobesFlux. A demo video is also available as a good start for beginners to learn how to use MicrobesFlux.

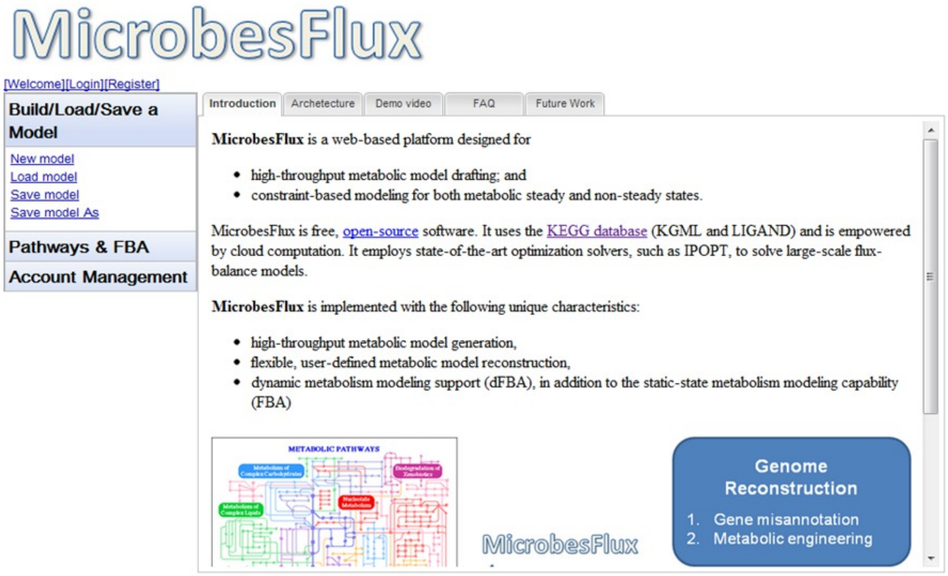

Figure 2: The landing page of MicrobesFlux

2.2 Register an account and login

Users are required to login to use MicrobesFlux. New users can click the "Register" link right below the MicrobesFlux logo and follow the instructions to register an account (Figure 3).

Registered users can login MicrobesFlux by clicking the "Login" link below the MicrobesFlux logo (Figure 4).

In order to change his/her password, a user can login to MicrobesFlux and click the Account Management stack to input the new password (Figure 5).

2.3 Troubleshooting

Question: Why do I see an "you have to log in to create a new model" error when I click "New Model"?
Answer: In MicrobesFlux, a user can save his/her customized model and get optimization results sent to his/her email address. In order to do that, MicrobesFlux needs to associate models to user accounts. Thus, a user has to log in to MicrobesFlux first.

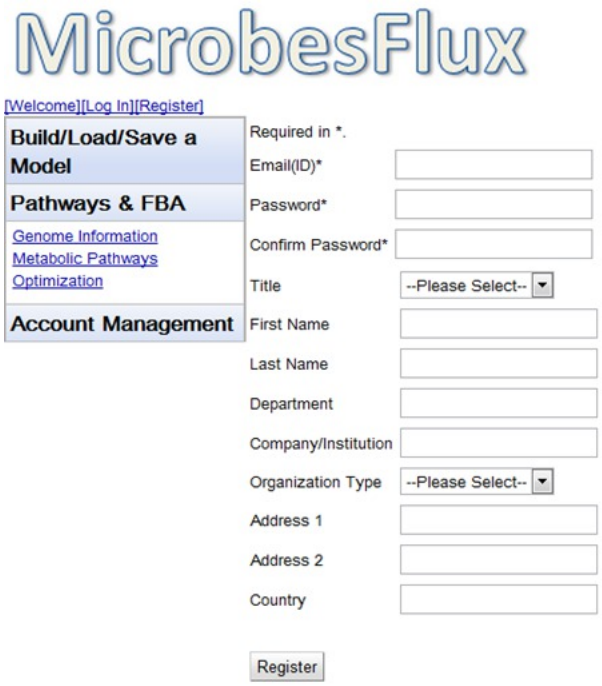

Figure 3: User registrations form in MicrobesFlux

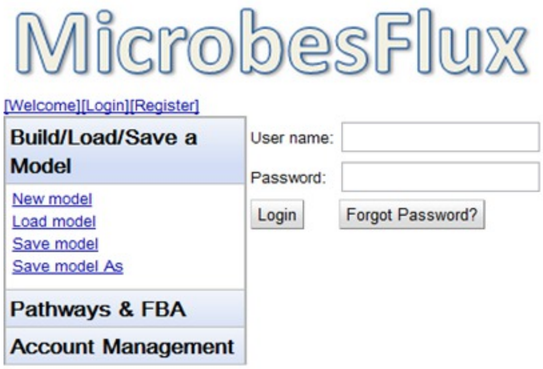

Figure 4: User login form in MicrobesFlux

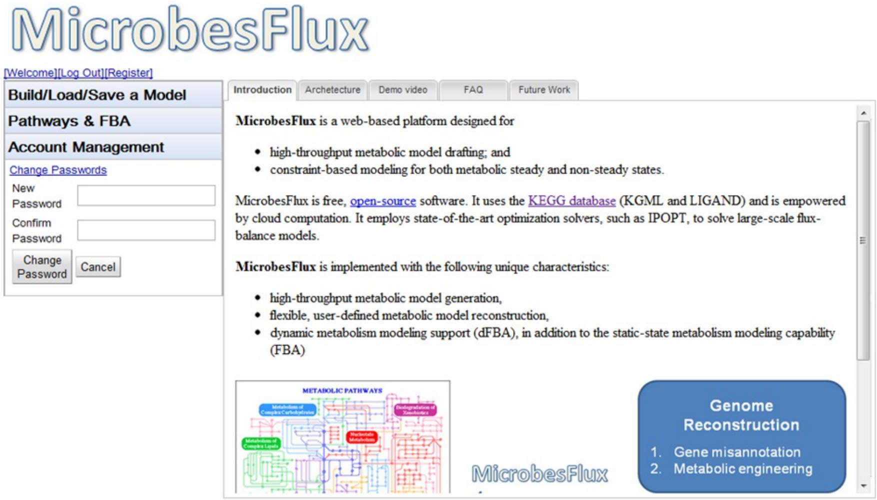

Figure 5: Changing passwords in MicrobesFlux

3 Model reconstruction from KEGG

3.1 Create a new model

In order to generate a seed model for the interested organism, click the New model link in the Build/Load/Save a Model stack. You can give a name to the model so that it can be saved and loaded in the future. An email address is needed for getting the various model results (e.g. SBML file of reconstructed models). In the "Input KEGG Organisms" box, type the three-letter organism code (e.g. eco for *Escherichia coli* K- 12 MG1655) or the organism name. Click "Run" to start reconstructing the metabolic model (Figure 6).

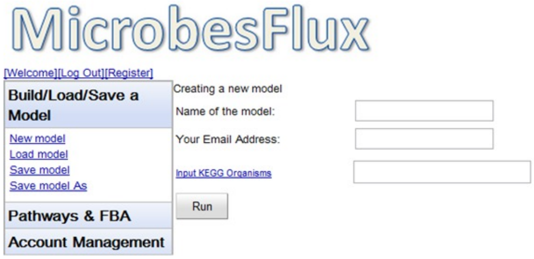

Figure 6: Creating a model in MicrobesFlux

If you are not sure about the name of your organisms, click the link of "Input KEGG Organisms". The link will lead you to the list of organisms in the KEGG database (Figure 7).

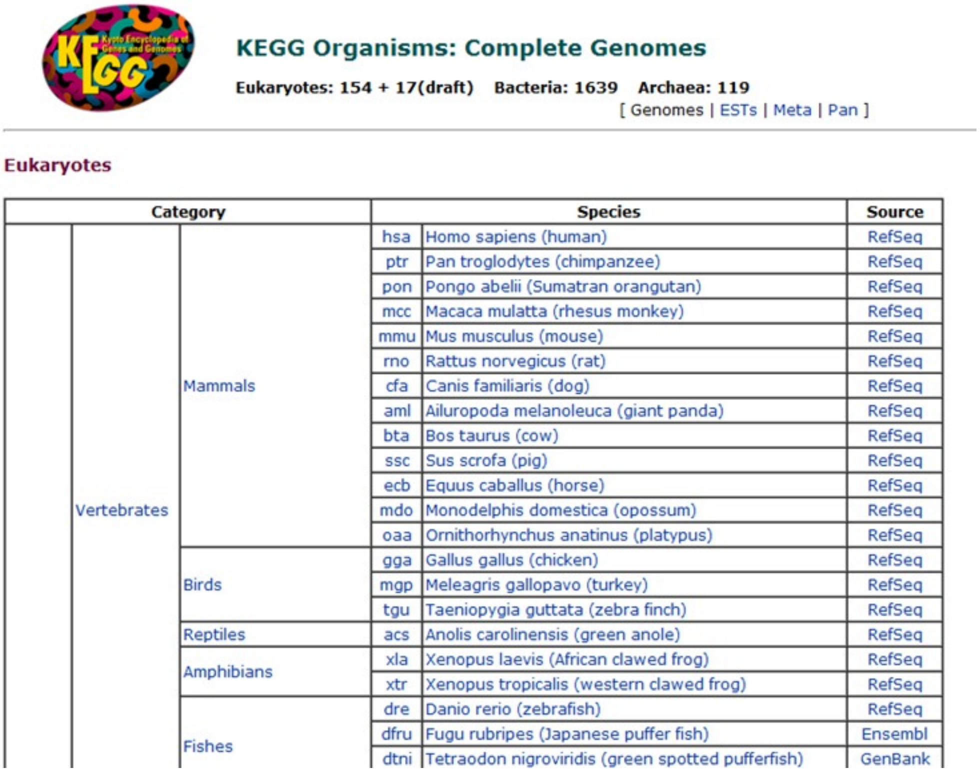

KEGG Organisms: Complete Genomes

Eukaryotes: 154 + 17(draft) Bacteria: 1639 Archaea: 119

[Genomes | ESTs | Meta | Pan]

Eukaryotes

Category			Species		Source
	Vertebrates	Mammals	hsa	Homo sapiens (human)	RefSeq
			ptr	Pan troglodytes (chimpanzee)	RefSeq
			pon	Pongo abelii (Sumatran orangutan)	RefSeq
			mcc	Macaca mulatta (rhesus monkey)	RefSeq
			mmu	Mus musculus (mouse)	RefSeq
			rno	Rattus norvegicus (rat)	RefSeq
			cfa	Canis familiaris (dog)	RefSeq
			aml	Ailuropoda melanoleuca (giant panda)	RefSeq
			bta	Bos taurus (cow)	RefSeq
			ssc	Sus scrofa (pig)	RefSeq
			ecb	Equus caballus (horse)	RefSeq
			mdo	Monodelphis domestica (opossum)	RefSeq
			oaa	Ornithorhynchus anatinus (platypus)	RefSeq
		Birds	gga	Gallus gallus (chicken)	RefSeq
			mgp	Meleagris gallopavo (turkey)	RefSeq
			tgu	Taeniopygia guttata (zebra finch)	RefSeq
		Reptiles	acs	Anolis carolinensis (green anole)	RefSeq
		Amphibians	xla	Xenopus laevis (African clawed frog)	RefSeq
			xtr	Xenopus tropicalis (western clawed frog)	RefSeq
		Fishes	dre	Danio rerio (zebrafish)	RefSeq
			dfru	Fugu rubripes (Japanese puffer fish)	Ensembl
			dtni	Tetraodon nigroviridis (green spotted pufferfish)	GenBank

Figure 7: Organisms list in KEGG database

3.2 Save & load a model

In order to save a model, simply click the "Save model" link whenever you feel like. If you wish to save the model with another name, click "Save model As" (Figure 8).

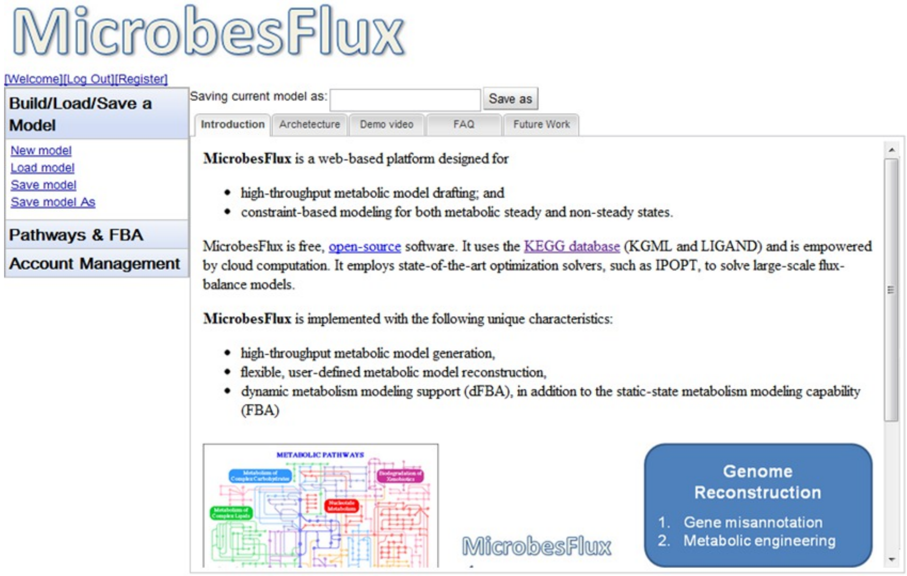

Figure 8: Save models using a new name

In order to load a model that is previously saved, click the "Load model" link. A list of saved models can be found in the drop down list (Figure 9). Choose the one that you want to load and click the "Load" button.

3.3 Troubleshooting

Question 1: Why do I see a page showing "Finding records that match your criteria"?
Answer: It happens when there is a communication problem between the server and the client. Users can simply refresh the webpage and re-login to load the same model and continue working on it without losing any data.

Question 2: Why do I get the window alert (Figure 10) about saving the current model before loading another one?
Answer: The window alert is a simple reminder for you to save the current model. If you have already saved the model or want to discard changes to the current model without saving it, the current model, click "OK" to proceed. Otherwise, click the "Cancel" button and MicrobesFlux will keep your current model intact.

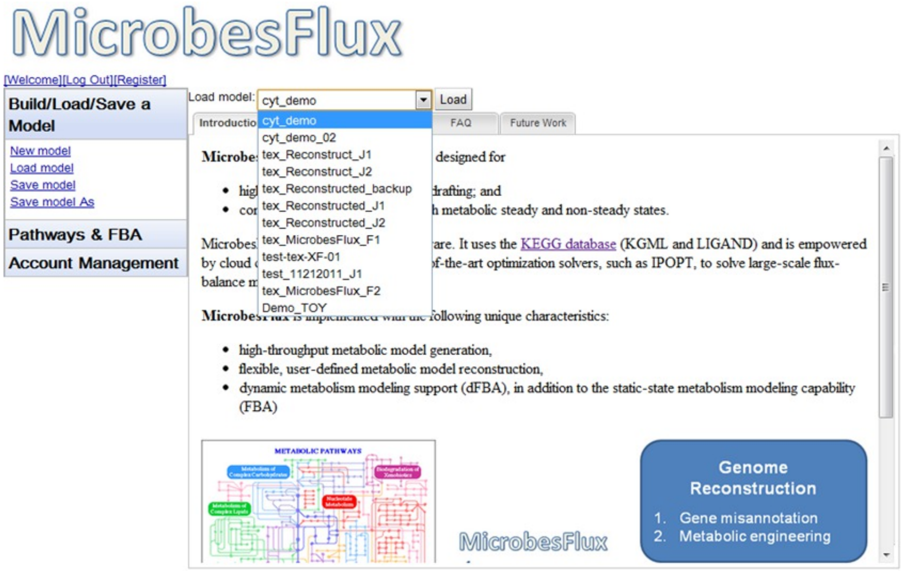

Figure 9: Load a model

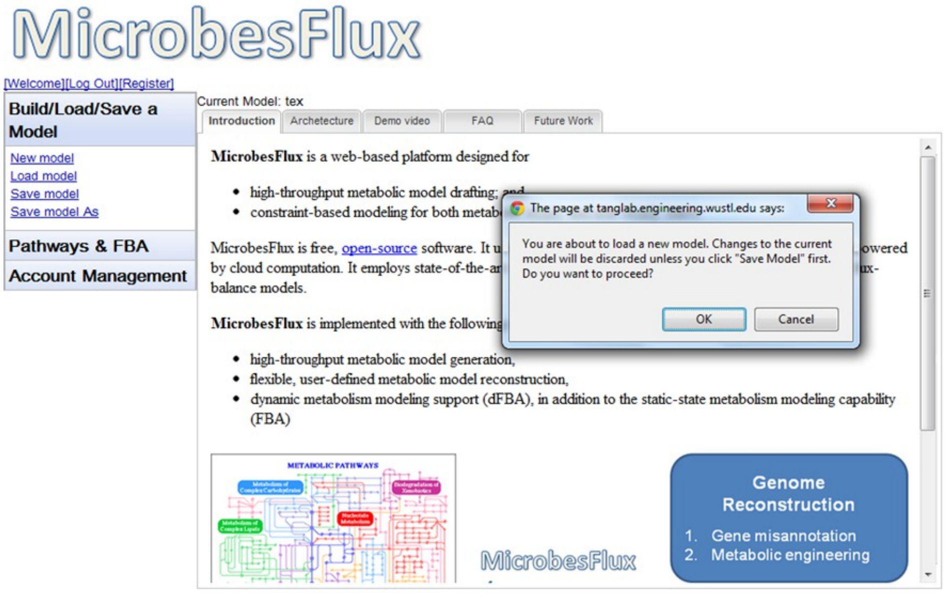

Figure 10: Window alert during model loading

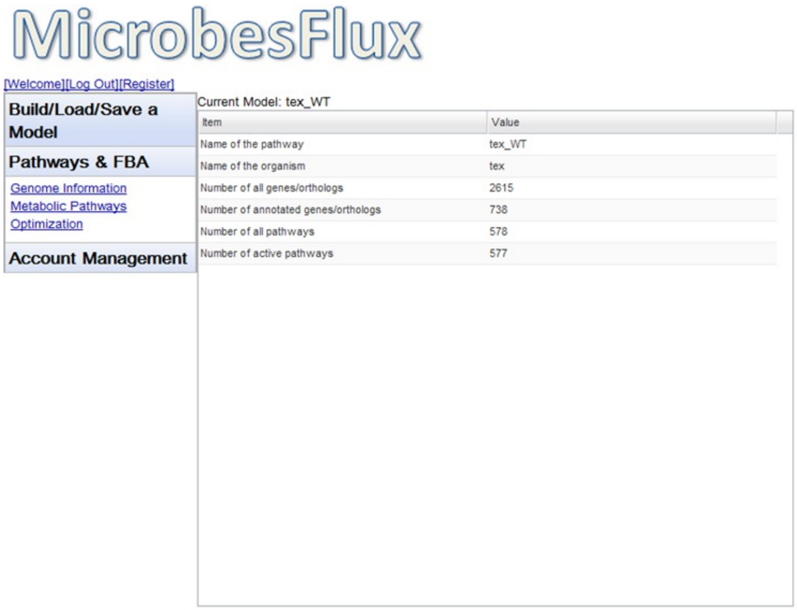

Figure 11: Genome information of a metabolic model

4 Metabolic models drafting

4.1 Genome information

After generating a seed model or load a previous model loaded in, the genome information of the model can be viewed by clicking "Genome Information" link in the "Pathway & FBA" stack (Figure 11). The number of genes and annotated genes, as well as the number of pathways and active pathways are calculated based on the genome sequencing stored in KGML files of the designated organisms.

4.2 Metabolic pathways manipulations

The metabolic pathways of the designated metabolic model can be viewed and manipulated by clicking "Metabolic Pathways" in the "Pathways & FBA" stack (Figure 12).

Users can choose to regroup metabolic reactions based on the metabolic reactions can be grouped by pathways (Figure 13a), reaction directions (i.e., arrows, Figure 13b), or other features such as pathway knock-out, reactants, and products.

To reconstruct a metabolic model, diverse tools are provided in MicrobesFlux, including 1) adding inflow/outflow reactions; 2) implementing biomass production reaction; 3) pathway knock-out; and 4) introducing heterologous pathways.

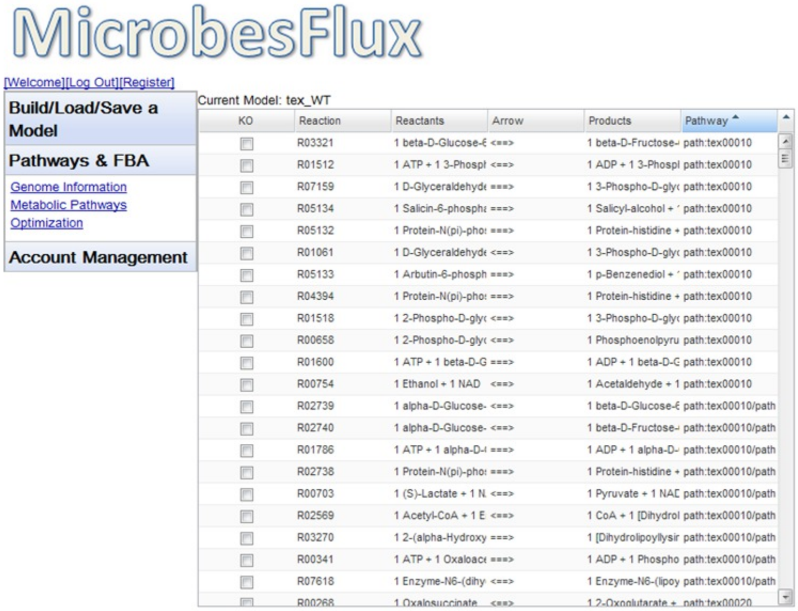

Figure 12: Pathways of a metabolic models

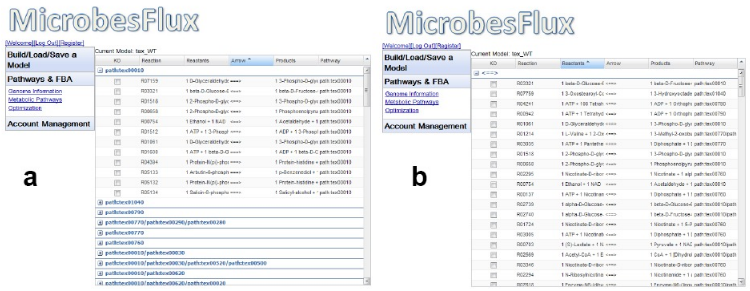

Figure 13: Grouping metabolic reactions

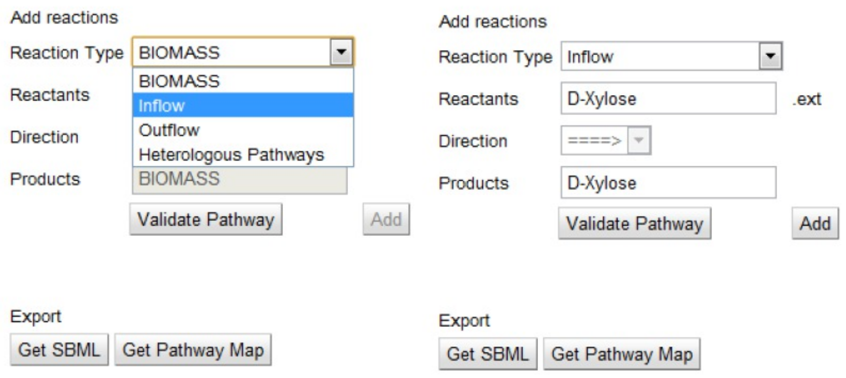

Figure 14: Add reactions to a pathway

4.3 Adding inflow/outflow reactions

The inflow/outflow reactions are the transport reactions between two metabolic compartments, cytosol and extracellular medium. These inflow/outflow reactions are determinant for building metabolic models. However, they are not originally included in the seed metabolic model generated from KGML files and KEGG LIGAND database.

To add inflow/outflow reactions in MicrobesFlux, in the Add reactions module, choose the reaction type as inflow (outflow), then put the metabolites that are exchanged between cytosol and the extracellular medium (Figure 4.4). The reactant will have the ".ext" suffix for inflow reactions that transport metabolite from extracellular medium to cytosol. The products will have the suffix ".ext" for outflow reactions that transport metabolite from cytosol to extracellular medium.

To upload the added inflow/outflow reactions into the metabolic model, click Validate Pathway button first to make sure that the names of metabolites are consistent with

with metabolites names in the MicrobesFlux database. A complete list of compound names that are recognizable can be found in `http://tanglab.engineering.wustl.edu/media/valid_compounds.html`.
Once the validation is done and the metabolites names are valid, click the "Add" button will add the inflow/outflow reactions to the metabolic model(Figure 14).

4.4 Implement biomass reactions

The biomass production reaction can be manually added in a similar fashion as shown above. In general, choose Biomass in Reaction Type and put the biomass compositions in the reactants column (Figure 4.4). Then validate pathway and add it into the reconstructed model. *Note*: The biomass production reaction is necessary for FBA studies in "Optimization" module.

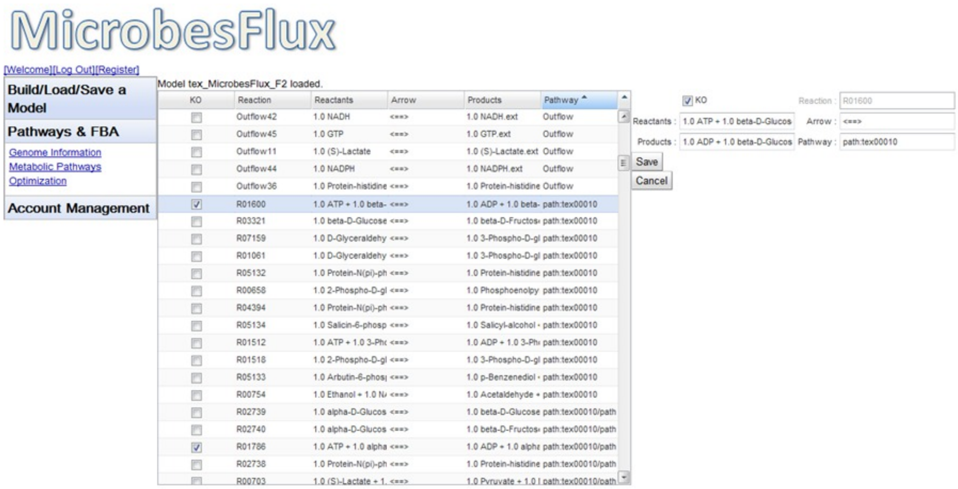

Figure 15: Knocking out pathways

4.5 Knocking-out and changing reactions from metabolic models

To manipulate a pathway, choose the designated reaction by clicking the reaction row in the column (Figure 15). A separate table will be present, which contains “Reactants”, “Products, “Arrows”, “Pathway”, and a “KO” box. Click the “KO” box to knock out a pathway, or change the reactants and products of the reaction as your will. The arrows of the reaction can also be switched between “<==>” and “===>”.

4.6 Export metabolic models

The metabolic model can be extracted in SBML format by clicking the “Get SBML” button (Figure **??**. Also, the metabolic model can be visualized in SVG format by clicking the “Get Pathway Map” button. The SBML file (Figure 17) and SVG (Figure 18) file will be sent to users email.

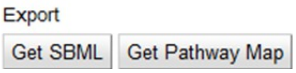

Figure 16: Export metabolic models

4.7 Troubleshooting

Question 1: Why the Add button cannot be activated?

```xml
<?xml version='1.0' encoding='UTF-8'?>
<sbml xmlns="http://www.sbml.org/sbml/level3/version1/core" level="3" version="1">
  <model id="tex_MicrobesFlux_F2">
    <listOfCompartments>
      <compartment id="cell" name="cell" constant="true"/>
    </listOfCompartments>
    <listOfSpecies>
      <species id="Cobaltprecorrin8" name="Cobalt-precorrin-8" compartment="cell" hasOnlySubstanceUnits="false" boundaryCondition="false" constant="false"/>
      <species id="LSeryltRNASer" name="L-Seryl-tRNA(Ser)" compartment="cell" hasOnlySubstanceUnits="false" boundaryCondition="false" constant="false"/>
      <species id="alphaDGlucose6phosphate" name="alpha-D-Glucose-6-phosphate" compartment="cell" hasOnlySubstanceUnits="false" boundaryCondition="false"
constant="false"/>
      <species id="DXylulose" name="D-Xylulose" compartment="cell" hasOnlySubstanceUnits="false" boundaryCondition="false" constant="false"/>
      <species name="5-O-(1-Carboxyvinyl)-3-phosphoshikimate" compartment="cell" hasOnlySubstanceUnits="false" boundaryCondition="false" constant="false"/>
      <species id="Cobaltprecorrin7" name="Cobalt-precorrin-7" compartment="cell" hasOnlySubstanceUnits="false" boundaryCondition="false" constant="false"/>
      <species id="Cobaltprecorrin6" name="Cobalt-precorrin-6" compartment="cell" hasOnlySubstanceUnits="false" boundaryCondition="false" constant="false"/>
      <species id="Cobaltprecorrin4" name="Cobalt-precorrin-4" compartment="cell" hasOnlySubstanceUnits="false" boundaryCondition="false" constant="false"/>
      <species id="LTyrosyltRNATyr" name="L-Tyrosyl-tRNA(Tyr)" compartment="cell" hasOnlySubstanceUnits="false" boundaryCondition="false" constant="false"/>
      <species id="Deoxycytidine" name="Deoxycytidine" compartment="cell" hasOnlySubstanceUnits="false" boundaryCondition="false" constant="false"/>
      <species name="LL-2,6-Diaminoheptanedioate" compartment="cell" hasOnlySubstanceUnits="false" boundaryCondition="false" constant="false"/>
      <species id="UndecaprenyldiphosphoNacetylmuramoylLalanylDglutamylmeso" name="Undecaprenyl-diphospho-N-acetylmuramoyl-L-alanyl-D-glutamyl-meso-"
compartment="cell" hasOnlySubstanceUnits="false" boundaryCondition="false" constant="false"/>
      <species name="HCO3-.ext" compartment="cell" hasOnlySubstanceUnits="false" boundaryCondition="false" constant="false"/>
      <species id="Selenocysteine" name="Selenocysteine" compartment="cell" hasOnlySubstanceUnits="false" boundaryCondition="false" constant="false"/>
      <species id="NAcetylDglucosamine" name="N-Acetyl-D-glucosamine" compartment="cell" hasOnlySubstanceUnits="false" boundaryCondition="false" constant="false"/>
      <species id="SuccinylCoA" name="Succinyl-CoA" compartment="cell" hasOnlySubstanceUnits="false" boundaryCondition="false" constant="false"/>
      <species id="C15813" name="C15813" compartment="cell" hasOnlySubstanceUnits="false" boundaryCondition="false" constant="false"/>
      <species id="C15810" name="C15810" compartment="cell" hasOnlySubstanceUnits="false" boundaryCondition="false" constant="false"/>
      <species id="C15814" name="C15814" compartment="cell" hasOnlySubstanceUnits="false" boundaryCondition="false" constant="false"/>
      <species id="C15815" name="C15815" compartment="cell" hasOnlySubstanceUnits="false" boundaryCondition="false" constant="false"/>
      <species id="NicotinateDribonucleotide" name="Nicotinate-D-ribonucleotide" compartment="cell" hasOnlySubstanceUnits="false" boundaryCondition="false"
constant="false"/>
      <species id="LArginine" name="L-Arginine" compartment="cell" hasOnlySubstanceUnits="false" boundaryCondition="false" constant="false"/>
      <species id="tRNATyr" name="tRNA(Tyr)" compartment="cell" hasOnlySubstanceUnits="false" boundaryCondition="false" constant="false"/>
      <species id="R3Hydroxyhexanoylacp" name="(R)-3-Hydroxyhexanoyl-[acp]" compartment="cell" hasOnlySubstanceUnits="false" boundaryCondition="false"
constant="false"/>
```


Figure 17: The SBML file of a metabolic model

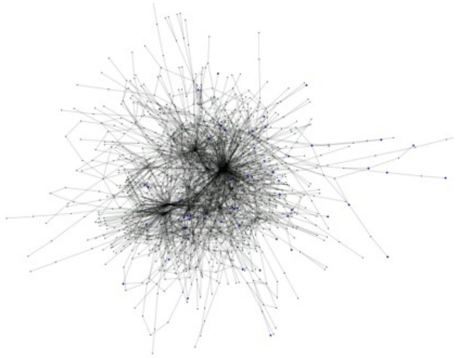

Figure 18: Metabolic network visualization (SVG)

Answer: Some of the metabolites in KEGG LIGAND database cannot be recognized by MicrobesFlux. A complete list of compound names that are recognizable can be found in `http://tanglab.engineering.wustl.edu/media/valid_compounds.html`.
In such case, we suggest users to use the KEGG Compound ID (e.g. C00001 for H_2O) instead.

Question 2: Do I have to input biomass production reaction manually?
Answer: Yes, the biomass production reaction has to be manually input in MicrobesFlux, based on two considerations.

First, the biomass compositions in different organisms are not the same. Therefore, a customized biomass production reaction entry is necessary.

Second, the completeness of biomass production reaction depends on the character of metabolic models. For a large-scale metabolic model, such as genome- scale metabolic model, the complete biomass composition including compositions of amino acids, lipids, nucleic acids, etc, is required. However, for a simplified metabolic model, it is not necessary to input all the biomass compositions into consideration. To cope with the diverse demands of metabolic models, we let users decide what should be input as biomass production reaction.

Question 3: How can I delete the inflow/outflow reactions?
Answer: Click the "KO" button before the inflow/outflow reactions to remove these reactions from the metabolic model.

5 Flux balance analysis (FBA) of metabolic models

The mathematical representation of FBA is

$$
\begin{aligned}
\min \sum_i \quad & c_i \ v_i \\
s.t. \qquad Sv \quad & = \quad 0 \\
lb \leq \quad v \quad & \leq vb
\end{aligned}
$$

This linear optimization can be solved by optimization solvers such as CPLEX and IPOPT, with the set-up of three modules: objective function, flux balance equations, and boundary conditions for each metabolic reaction. The flux balance equations will be automatically generated by MicrobesFlux, while the objective function and boundary conditions will need to be identified by users.

5.1 Designate the objective function

Two types of objective functions can be designated for flux balance analysis in MicrobesFlux: maximizing biomass, or maximizing a customer-defined objective function (Figure 19).

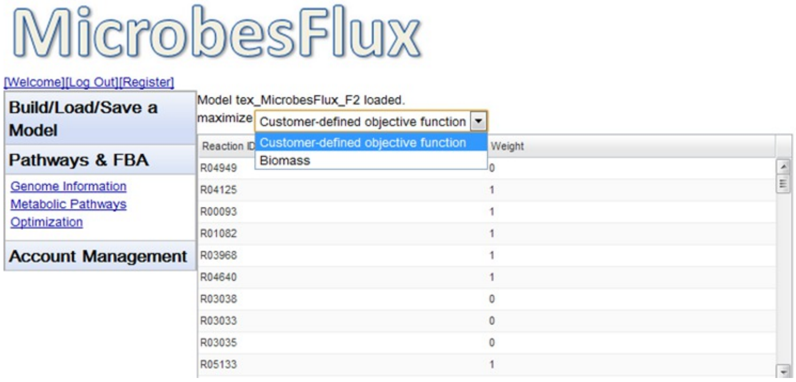

Figure 19: Setting up the objective function

If maximizing biomass is chosen as the objective function, please choose "Biomass" in the objective function column and make sure that the biomass production reaction has been included in the metabolic model (Figure 5.1). If maximizing a customer-defined objective function (i.e., max $\sum c_i v_i$) is chosen as the objective function, the weighting factor of each metabolic reaction can be manually tuned in the "Optimization" stack (Figure 20).

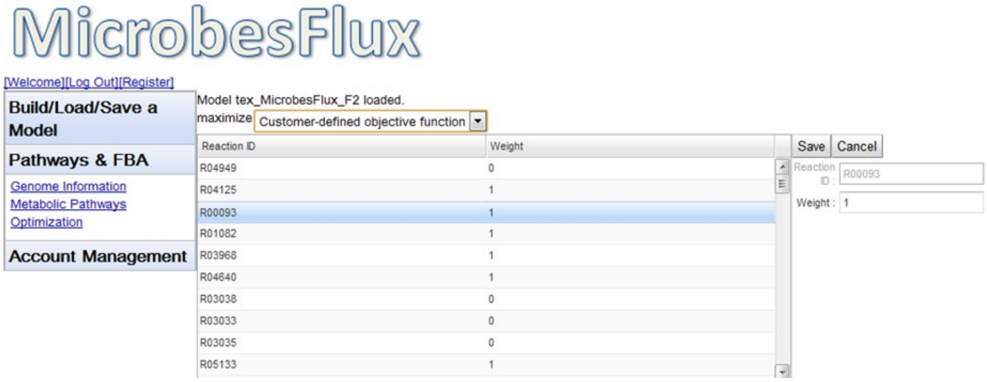

Figure 20: User-defined objective function

5.2 Generate flux balance equations

The flux balance equations (i.e., $Sv = 0$) are automatically generated by MicrobesFlux, based on the metabolic model reconstructed by users (Figure 21). To change the flux balance equations, users have to make new modifications in "Metabolic Pathways" module.

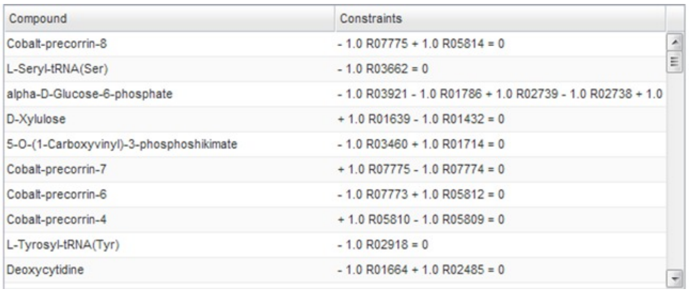

Compound	Constraints
Cobalt-precorrin-8	- 1.0 R07775 + 1.0 R05814 = 0
L-Seryl-tRNA(Ser)	- 1.0 R03662 = 0
alpha-D-Glucose-6-phosphate	- 1.0 R03921 - 1.0 R01786 + 1.0 R02739 - 1.0 R02738 + 1.0
D-Xylulose	+ 1.0 R01639 - 1.0 R01432 = 0
5-O-(1-Carboxyvinyl)-3-phosphoshikimate	- 1.0 R03460 + 1.0 R01714 = 0
Cobalt-precorrin-7	+ 1.0 R07775 - 1.0 R07774 = 0
Cobalt-precorrin-6	- 1.0 R07773 + 1.0 R05812 = 0
Cobalt-precorrin-4	+ 1.0 R05810 - 1.0 R05809 = 0
L-Tyrosyl-tRNA(Tyr)	- 1.0 R02918 = 0
Deoxycytidine	- 1.0 R01664 + 1.0 R02485 = 0

Figure 21: Flux balance equations

5.3 Set the boundary conditions

The boundary conditions of each metabolic flux can be set (shown in Figure 22). lb indicates the lower bound of the chosen reaction rate while ub indicates the upper bound of the chosen reaction rate. The lb cannot exceed ub. When $lb = ob$, the metabolic flux is effectively fixed to lb (or ub).

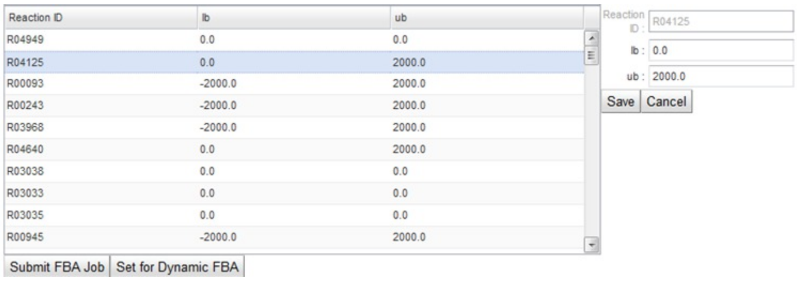

Reaction ID	lb	ub
R04949	0.0	0.0
R04125	0.0	2000.0
R00093	-2000.0	2000.0
R00243	-2000.0	2000.0
R03968	-2000.0	2000.0
R04640	0.0	2000.0
R03038	0.0	0.0
R03033	0.0	0.0
R03035	0.0	0.0
R00945	-2000.0	2000.0

Figure 22: Setting up boundary conditions

5.4 Submit an FBA job

Once the objective function is designated and the boundary conditions are set, click the "Submit FBA Job" to submit the request to MicrobesFlux for solving the FBA problem. It will normally take 1 5 minutes to get the email from MicrobesFlux, with the result file attached.

5.5 Dynamic flux balance analysis

MicrobesFlux also provide dynamic flux balance analysis (dFBA) for the same metabolic model reconstructed by users. Click the "Submit for Dynamic FBA" button to start the setting for dFBA in MicrobesFlux (Figure 23).

The dFBA in MicrobesFlux decomposes the dynamic microbial metabolism into numerous steady state metabolisms with various inflow/outflow fluxes (Figure 23). Therefore, an additional data file (in txt format) is needed to identify

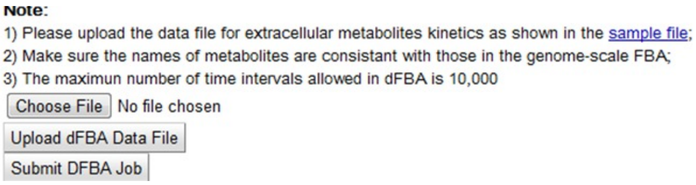

Figure 23: Dynamic flux balance analysis in MicrobesFlux

the inflow/outflow fluxes at each of the steady-state metabolism (Figure refdfba-data).

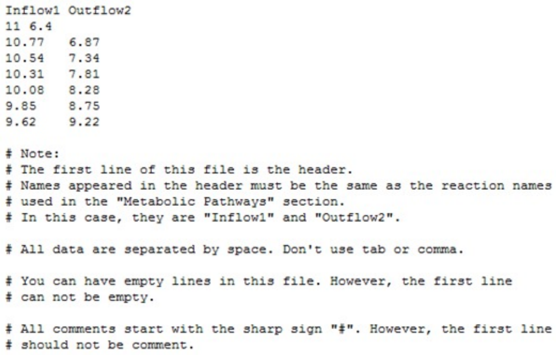

```
Inflow1 Outflow2
11 6.4
10.77   6.87
10.54   7.34
10.31   7.81
10.08   8.28
9.85    8.75
9.62    9.22

# Note:
# The first line of this file is the header.
# Names appeared in the header must be the same as the reaction names
# used in the "Metabolic Pathways" section.
# In this case, they are "Inflow1" and "Outflow2".

# All data are separated by space. Don't use tab or comma.

# You can have empty lines in this file. However, the first line
# can not be empty.

# All comments start with the sharp sign "#". However, the first line
# should not be comment.
```

Figure 24: A sample dFBA data used in MicrobesFlux

After uploading the data file to MicrobesFlux, click “Submit DFBA Job” to send the request for solving the dFBA problem. It will normally take one to five minutes for users to get the email from MicrobesFlux, with the result file attached.

5.6 Troubleshooting

Question 1: Why cannot I receive any email from MicrobesFlux?
Answer: Please check the spam box to see if there is any email. For dFBA job, please make sure that the data file uploaded follows the format as that shown in `http://tanglab.engineering.wustl.edu/media/sample.txt`. If there is still no email from MicrobesFlux, please contact us and report it as an issue using `http://code.google.com/p/kegg-dfba/`.

Question 2: Why the results of FBA/dFBA do not make any sense?
Answer: Please double-check the metabolic model reconstructed and makes sure that the gaps (such as the dead-end metabolic reaction, co-factor imbalance, etc) are filled in the reconstructed metabolic model. The protocol for generating a high- quality genome-scale metabolic reconstruction can be found in “Thiele I,

Palsson B∅. A protocol for generating a high-quality genome-scale metabolic reconstruction. Nat Protoc. 2010 Jan;5(1):93-121"

6 A case study in MicrobesFlux

Here we present a case study by using MicrobesFlux to reconstruct a medium-scale metabolic model for *Thermoanaerobacter* sp. strain X514. The drafted model is consisted of 196 metabolites and 229 reactions (162 intracellular reactions, 19 inflow/ outflow reactions, 39 gap-filling reactions, and 9 biomass-producing reactions). The major steps in metabolic model reconstruction are listed as below:

Step 1: login to MicorbesFlux and build a seed model. Choosing "tex *Thermoanaerobacter*_X514" as the organism (Figure 25).

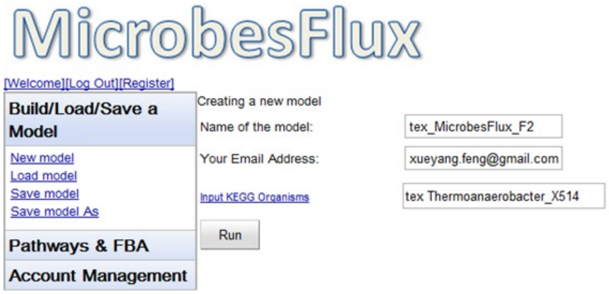

Figure 25: Build a seed model for *Thermoanaerobacter* sp. strain X514

Step 2: Input inflow/outflow fluxes in the reconstructed model, based on the literature survey of identified transporters in *Thermoanaerobacter* sp. strain X514 and gap-fill requirements of the model. To delete inflow/outflow fluxes, choose the reactions and mark as "KO" (Figure refinflow-outflow).

Step 3: Input biomass production reaction and heterologous pathways in the reconstructed model. Here, we use the following reaction for biomass production:

```
0.011 NAD + 0.009 NADP + 0.01 CoA + 0.016 Tetrahydrofolate +
0.528 C00017 + 0.066 C00046 + 0.026 C00039 + 0.076 C01356 +
0.101 C00889 + 0.08 C06707 + 0.043 C00182 + 220.0 ATP --> Biomass
```

The gaps in metabolic models can come from multiple sources. First, depends on the size and purpose of the metabolic model, some metabolic reactions need to be changed (e.g. Knock-out), which will be addressed in **Step 4**. Second, some metabolic reactions may be mis-annotated in the seed model or introduced as a foreign reaction from other biological systems. These pathway will be input in the reconstruction step as "Heterologous Pathways".

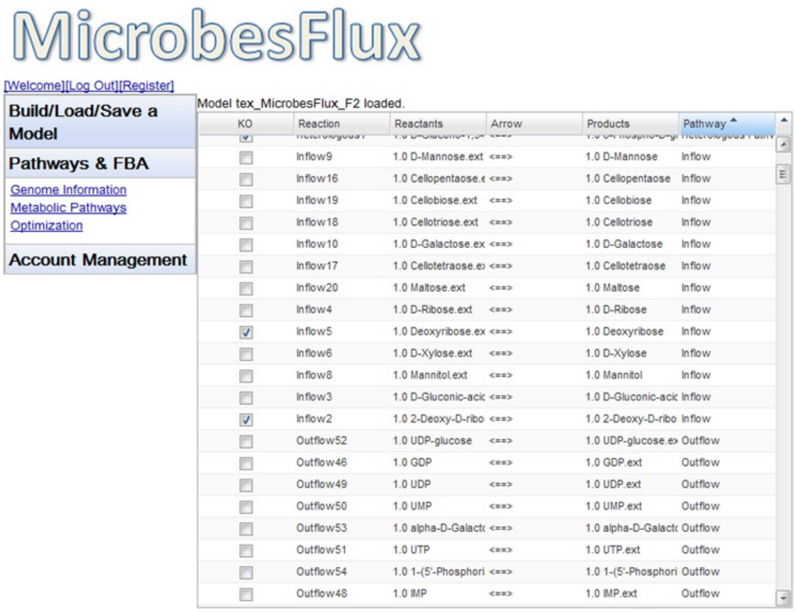

Figure 26: Input inflow/outflow fluxes in the reconstructed model of *Thermoanaerobacter* sp. strain X514

The KEGG Compound IDs are always suggested to be used due to its comparability with MicrobesFlux. Some compound, such as NADP can also be recognized in MicrobesFlux. A complete list of compound names that are recognizable can be found in `http://tanglab.engineering.wustl.edu/media/valid_compounds.html`.

Step 4: Manipulate pathways in the metabolic model. Since the draft model is a simplified model focusing on carbohydrate metabolism and amino acids biosynthesis, pathways that are not of interest in the reconstructed model can be deleted by marking the pathway as "KO". The cofactors involved in the reactions and the pathway directions can also be manipulated (Figure 28).

Step 5: Output the reconstructed model as SBML. Once the reconstruction of metabolic model is finished, the SBML file can be generated. The SBML files can then be read and used by other fluxomics software packages (Figure 29).

Step 6: Set the objective functions and boundary conditions for FBA. Here, we choose "maximize biomass" as the objective function, and set the boundary conditions for each metabolic flux. Certain inflow/outflow fluxes are needed to be assigned with fixed values.

Step 7: Get the result file of the reconstructed model. The result file will include three parts: 1) Genome Info; 2) Pathways and 3) AMPL file of the reconstructed model. In the AMPL file, the objective function, flux balance equations and the boundary conditions are included. The simulation results can be found in the "Results" section of AMPL file.

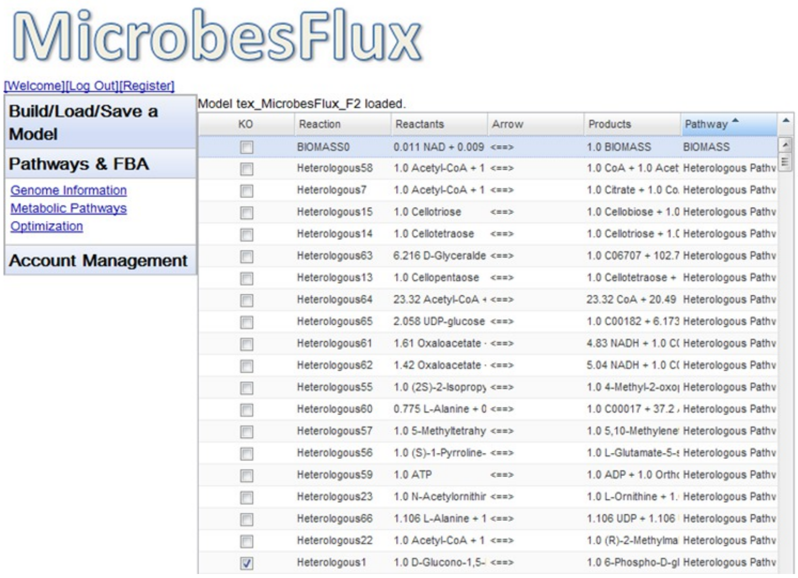

Figure 27: Input biomass production reaction in the reconstructed model of *Thermoanaerobacter* sp. strain X514

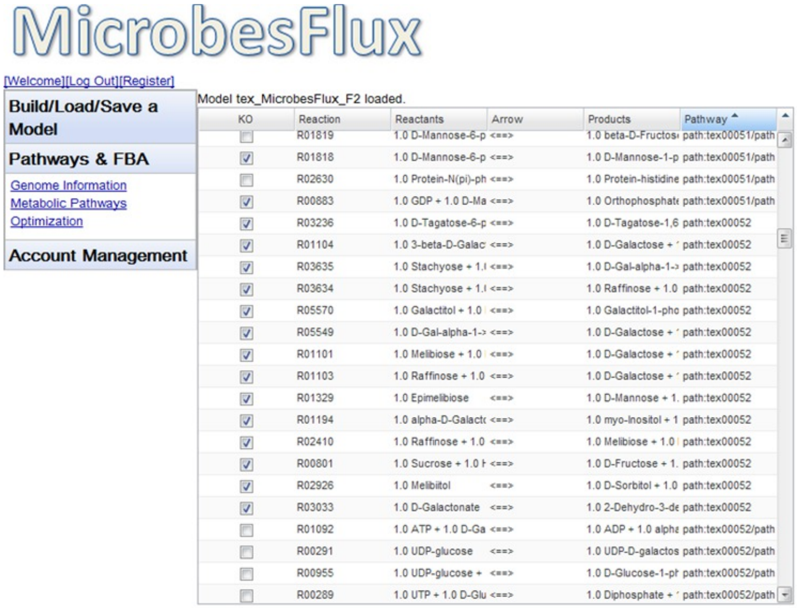

Figure 28: Manipulate pathways in the in the reconstructed model of *Thermoanaerobacter* sp. strain X514

```
▼<sbml xmlns="http://www.sbml.org/sbml/level3/version1/core" level="3" version="1">
  ▼<model id="tex_MicrobesFlux_F2">
    ▼<listOfCompartments>
        <compartment id="cell" name="cell" constant="true"/>
      </listOfCompartments>
    ▼<listOfSpecies>
        <species id="Cobaltprecorrin8" name="Cobalt-precorrin-8" compartment="cell" hasOnlySubstanceUnits="false" boundaryCondition="false" constant="false"/>
        <species id="LSeryltRNASer" name="L-Seryl-tRNA(Ser)" compartment="cell" hasOnlySubstanceUnits="false" boundaryCondition="false" constant="false"/>
        <species id="alphaDGlucose6phosphate" name="alpha-D-Glucose-6-phosphate" compartment="cell" hasOnlySubstanceUnits="false" boundaryCondition="false"
        constant="false"/>
        <species id="DXylulose" name="D-Xylulose" compartment="cell" hasOnlySubstanceUnits="false" boundaryCondition="false" constant="false"/>
        <species name="5-O-(1-Carboxyvinyl)-3-phosphoshikimate" compartment="cell" hasOnlySubstanceUnits="false" boundaryCondition="false" constant="false"/>
        <species id="Cobaltprecorrin7" name="Cobalt-precorrin-7" compartment="cell" hasOnlySubstanceUnits="false" boundaryCondition="false" constant="false"/>
        <species id="Cobaltprecorrin6" name="Cobalt-precorrin-6" compartment="cell" hasOnlySubstanceUnits="false" boundaryCondition="false" constant="false"/>
        <species id="Cobaltprecorrin4" name="Cobalt-precorrin-4" compartment="cell" hasOnlySubstanceUnits="false" boundaryCondition="false" constant="false"/>
        <species id="LTyrosyltRNATyr" name="L-Tyrosyl-tRNA(Tyr)" compartment="cell" hasOnlySubstanceUnits="false" boundaryCondition="false" constant="false"/>
        <species id="Deoxycytidine" name="Deoxycytidine" compartment="cell" hasOnlySubstanceUnits="false" boundaryCondition="false" constant="false"/>
        <species name="LL-2,6-Diaminoheptanedioate" compartment="cell" hasOnlySubstanceUnits="false" boundaryCondition="false" constant="false"/>
        <species id="UndecaprenyldiphosphoNacetylmuramoylLalanylDglutamylmeso" name="Undecaprenyl-diphospho-N-acetylmuramoyl-L-alanyl-D-glutamyl-meso-"
        compartment="cell" hasOnlySubstanceUnits="false" boundaryCondition="false" constant="false"/>
        <species name="HCO3-.ext" compartment="cell" hasOnlySubstanceUnits="false" boundaryCondition="false" constant="false"/>
        <species id="Selenocysteine" name="Selenocysteine" compartment="cell" hasOnlySubstanceUnits="false" boundaryCondition="false" constant="false"/>
        <species id="NAcetylDglucosamine" name="N-Acetyl-D-glucosamine" compartment="cell" hasOnlySubstanceUnits="false" boundaryCondition="false" constant="false"/>
        <species id="SuccinylCoA" name="Succinyl-CoA" compartment="cell" hasOnlySubstanceUnits="false" boundaryCondition="false" constant="false"/>
        <species id="C15813" name="C15813" compartment="cell" hasOnlySubstanceUnits="false" boundaryCondition="false" constant="false"/>
        <species id="C15810" name="C15810" compartment="cell" hasOnlySubstanceUnits="false" boundaryCondition="false" constant="false"/>
        <species id="C15814" name="C15814" compartment="cell" hasOnlySubstanceUnits="false" boundaryCondition="false" constant="false"/>
        <species id="C15815" name="C15815" compartment="cell" hasOnlySubstanceUnits="false" boundaryCondition="false" constant="false"/>
        <species id="NicotinateDribonucleotide" name="Nicotinate-D-ribonucleotide" compartment="cell" hasOnlySubstanceUnits="false" boundaryCondition="false"
        constant="false"/>
        <species id="LArginine" name="L-Arginine" compartment="cell" hasOnlySubstanceUnits="false" boundaryCondition="false" constant="false"/>
        <species id="tRNATyr" name="tRNA(Tyr)" compartment="cell" hasOnlySubstanceUnits="false" boundaryCondition="false" constant="false"/>
        <species id="R3Hydroxyhexanoylacp" name="(R)-3-Hydroxyhexanoyl-[acp]" compartment="cell" hasOnlySubstanceUnits="false" boundaryCondition="false"
        constant="false"/>
        <species name="2-Methylmaleate" compartment="cell" hasOnlySubstanceUnits="false" boundaryCondition="false" constant="false"/>
        <species name="(2S)-2-Isopropyl-3-oxosuccinate" compartment="cell" hasOnlySubstanceUnits="false" boundaryCondition="false" constant="false"/>
        <species name="3-Mercaptolactate" compartment="cell" hasOnlySubstanceUnits="false" boundaryCondition="false" constant="false"/>
```


Figure 29: SBML files for the reconstructed model of *Thermoanaerobacter* sp. strain X514

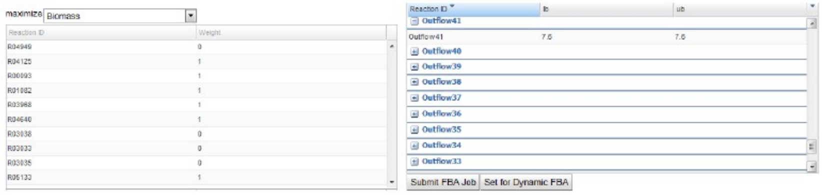

Figure 30: FBA setting for the reconstructed model of *Thermoanaerobacter* sp. strain X514

```
Model created: 2012-01-04T14:37:14.195855
======================================
========  Part I: Genome Info ========
======================================
Name of the pathway: tex_Reconstruct_J1
Name of the organism: tex
Number of all genes/orthologs: 2615
Number of all genes/orthologs: 2615
Number of annotated genes/orthologs: 738
Number of all pathways: 645
Number of active pathways: 645
======================================
========   Part II: Pathways =========
--------------------------------------
 X R04949 : 1.0 Cyanoglycoside + 1.0 H2O <--> 1.0 Cyanohydrin + 1.0 D-Glucose
   R04125 : 1.0 S-Aminomethyldihydrolipoylprotein + 1.0 Tetrahydrofolate <--> 1.0 Dihydrolipoylprotein + 1.0 5,10-Methylenetetrahydrofolate + 1.0 NH3
   R00093 : 2.0 L-Glutamate + 1.0 NAD <--> 1.0 L-Glutamine + 1.0 2-Oxoglutarate + 1.0 NADH
   R01082 : 1.0 (S)-Malate <--> 1.0 Fumarate
   R03968 : 1.0 (2S)-2-Isopropylmalate <--> 1.0 2-Isopropylmaleate
   R04640 : 1.0 5-(5-Phospho-D-ribosylaminoformimino)-1-(5-phosphoribosyl)- <--> 1.0 N-(5'-Phospho-D-1'-ribulosylformimino)-5-amino-1-(5''-phospho-D-
 X R03038 : 1.0 ATP + 1.0 L-Alanine + 1.0 tRNA(Ala) <--> 1.0 AMP + 1.0 Diphosphate + 1.0 L-Alanyl-tRNA
 X R03033 : 1.0 D-Galactonate <--> 1.0 2-Dehydro-3-deoxy-D-galactonate + 1.0 H2O
 X R03035 : 1.0 ATP + 1.0 Pantetheine-4'-phosphate <--> 1.0 Diphosphate + 1.0 Dephospho-CoA
   R05133 : 1.0 Arbutin-6-phosphate <--> 1.0 p-Benzenediol + 1.0 beta-D-Glucose-6-phosphate
 X R00428 : 1.0 GTP + 1.0 H2O <--> 1.0 Formamidopyrimidine-nucleoside-triphosphate
   R00586 : 1.0 L-Serine + 1.0 Acetyl-CoA <--> 1.0 O-Acetyl-L-serine + 1.0 CoA
 X R05807 : 1.0 Sirohydrochlorin + 1.0 Cobalt-ion <--> 1.0 Cobalt-sirohydrochlorin + 2.0 H
 X R00425 : 1.0 GTP + 3.0 H2O <--> 1.0 Formate + 1.0 2,5-Diamino-6-(5'-phosphoribosylamino)-4-pyrimidineone + 1.0 Diphosphate
 X R06447 : 1.0 trans,trans,cis-Geranylgeranyl-diphosphate + 7.0 Isopentenyl-diphosphate <--> 1.0 di-trans,poly-cis-Undecaprenyl-diphosphate + 7.0 Diphos
   R01641 : 1.0 Sedoheptulose-7-phosphate + 1.0 D-Glyceraldehyde-3-phosphate <--> 1.0 D-Ribose-5-phosphate + 1.0 D-Xylulose-5-phosphate
 X R04544 : 1.0 (3R)-3-Hydroxypalmitoyl-[acyl-carrier-protein] <--> 1.0 trans-Hexadec-2-enoyl-[acp] + 1.0 H2O
 X R06529 : 1.0 Adenosyl-cobyrinate-hexaamide + 1.0 D-1-Aminopropan-2-ol-O-phosphate + 1.0 ATP <--> 1.0 Adenosyl-cobinamide-phosphate + 1.0 ADP + 1.0 Ort
 X R04543 : 1.0 (3R)-3-Hydroxypalmitoyl-[acyl-carrier-protein] + 1.0 NADP <--> 1.0 3-Oxohexadecanoyl-[acp] + 1.0 NADPH + 1.0 H
 X R04463 : 1.0 ATP + 1.0 5'-Phosphoribosyl-N-formylglycinamide + 1.0 L-Glutamine + 1.0 H2O <--> 1.0 ADP + 1.0 Orthophosphate + 1.0 2-(Formamido)-N1-(5'-
phosphoribosyl)acetamidine + 1.0 L-Glutamate
   R02291 : 1.0 L-Aspartate-4-semialdehyde + 1.0 Orthophosphate + 1.0 NADP <--> 1.0 4-Phospho-L-aspartate + 1.0 NADPH
   R05134 : 1.0 Salicin-6-phosphate <--> 1.0 Salicyl-alcohol + 1.0 beta-D-Glucose-6-phosphate
   R04394 : 1.0 Protein-N(pi)-phospho-L-histidine + 1.0 Salicin <--> 1.0 Protein-histidine + 1.0 Salicin-6-phosphate
 X R02295 : 1.0 Nicotinate-D-ribonucleoside + 1.0 Orthophosphate <--> 1.0 Nicotinate + 1.0 alpha-D-Ribose-1-phosphate + 1.0 H
======== Results =========

Ipopt 3.8.3: Optimal Solution Found

suffix ipopt_zU_out OUT;
suffix ipopt_zL_out OUT;
 === Flux Results ===
Objective function value 0.042040
R04125   ->      0.204045
R00093   ->      -782.108612
BIOMASS0         ->      0.042040
R05133   ->      0.000000
R05132   ->      0.000000
R00946   ->      0.017380
R05134   ->      0.000000
Inflow16         ->      0.000000
Inflow17         ->      0.000000
R00228   ->      -3.583762
R04001   ->      -0.009522
R03968   ->      0.009522
R01827   ->      0.002073
R00226   ->      861.455332
R00220   ->      0.149241
R04640   ->      0.003241
R02283   ->      -0.002952
Outflow41        ->      7.600000
Outflow40        ->      316.446412
Outflow43        ->      -780.867679
Outflow42        ->      -7.600462
Outflow45        ->      -389.141926
Outflow44        ->      780.867301
Heterologous7    ->      0.035584
Outflow46        ->      389.141926
Outflow49        ->      0.015855
Outflow48        ->      -389.141926
R00014   ->      867.567233
R01015   ->      -3.891836
R00945   ->      0.186665
```

Figure 31: Result file of FBA studies for reconstructed model of *Thermoanaerobacter* sp. strain X514
